# Prevalence of girl and boy child marriage across states and Union Territories in India, 1993–2021: a repeated cross-sectional study

**DOI:** 10.1016/S2214-109X(23)00470-9

**Published:** 2023-12-15

**Authors:** Jewel Gausman, Rockli Kim, Akhil Kumar, Shamika Ravi, S V Subramanian

**Affiliations:** a Department of Global Health and Population, Harvard T H Chan School of Public Health, Boston, MA, USA; b Department of Social and Behavioral Sciences, Harvard T H Chan School of Public Health, Boston, MA, USA; c Guttmacher Institute, New York, NY, USA; d Maternal and Child Health Nursing Department, School of Nursing, University of Jordan, Amman, Jordan; e Division of Health Policy and Management, College of Health Science, Korea University, Seoul, South Korea; f Interdisciplinary Program in Precision Public Health, Department of Public Health Sciences, Korea University, Seoul, South Korea; g Center for Geographic Analysis, Harvard University, Boston, MA, USA; h University of Toronto, Toronto, ON, Canada; i Government of India, New Delhi, India; j Harvard Center for Population and Development Studies, Cambridge, MA, USA

## Abstract

**Background:**

India's success in eliminating child marriage is crucial to the achievement of the Sustainable Development Goal target 5.3. We aimed to estimate the prevalence of child marriage in girls and boys in India and describe its change across 36 states and Union Territories between 1993 and 2021.

**Methods:**

For this cross-sectional study, data from five National Family Health Surveys from 1993, 1999, 2006, 2016, and 2021 were used. The study included 310 721 women aged 20–24 years between 1993 and 2021 and 43 436 men aged 20–24 years between 2006 and 2021. Child marriage was defined as marriage in individuals younger than 18 years for men and women. We calculated the annual change in prevalence during the study period for states and Union Territories and estimated the population headcount of child brides and grooms.

**Findings:**

Child marriage declined during 1993 to 2021. The all-India prevalence of child marriage in girls declined from 49·4% (95% CI 48·1–50·8) in 1993 to 22·3% (21·9–22·7) in 2021. Child marriage in boys declined from 7·1% (6·9–30·8) in 2006 to 2·2% (1·8–2·7) in 2021. The largest decreases in child marriage occurred between 2006 and 2016. Between 2016 and 2021, a few states and Union Territories saw an increase in prevalence of child marriage in girls (n=6) and boys (n=8) despite declines in the all-India prevalence. In 2021, 13 464 450 women aged 20–24 years and 1 454 894 men aged 20–24 years were estimated to be married as children.

**Interpretation:**

One in five girls and nearly one in six boys are still married below the legal age of marriage in India. There remains an urgent need for strengthened national and state-level policy to eliminate child marriage by 2030.

**Funding:**

Bill & Melinda Gates Foundation.

## Introduction

Child marriage is a human rights violation and a recognised form of sexual and gender-based violence. Defined as marriage under the age of 18 years, child marriage is both a cause and consequence of social and economic vulnerability that leads to a range of poor health consequences that limit the ability of boys and girls to reach their full potential.^1^, ^2^ Although child marriage in girls receives more attention, child marriage in boys is gaining recognition as a global concern.

The Sustainable Development Goal (SDG) target 5.3 aims to end child marriage in girls by 2030 as part of a global commitment to “eliminate all harmful practices, such as child, early and forced marriage and female genital mutilation”. For girls, the annual rate of reduction in the prevalence of child marriage must increase from 1·9% to 23% globally to meet the SDG target.^3^ Globally, child marriage in boys is thought to have declined during the last 25 years, although, it is not specifically recognised by the SDG targets. A substantial percentage of boys are affected by the practice globally, and there is wide variability in the prevalence across countries ranging from less than 1% to as high as 30%.^4^ The global hotspots for child marriage in girls differ from the settings with a high prevalence of the practice among boys.^4^

National governments, especially those in south Asia, have implemented a wide variety of programmes and policies geared to target the underlying social and structural drivers of child marriage with mixed success.^5^ Understanding sub-national variation over time of child marriage has been identified as an important research priority to better focus investments and address inequalities in the rate of change.^6^

India's success is crucial in achieving the SDG target 5.3.^7^ The national rate of decline in child marriage during the last three decades has been considerable; however, previous research suggests that substantial variability of the rate of decline of child marriage at the sub-national level exists.^8^ Given that state governments tend to enact social sector policy in India, historical implementation of programmes to address child marriage has varied across and within states.^8^ Further, Indian states have rich and varied histories that have resulted in large differences in population health, economic development, and education, which are many of the underlying drivers of child marriage.

In this study, we aim to present a systematic description of the trends in child marriage in girls and boys aged 20–24 years in India and its 36 states and Union Territories between 1993 and 2021. The age range considered corresponds to the SDG target 5.3, which aims to eliminate the practice.

**Research in context**

**Evidence before this study**

We searched PubMed and JSTOR on April 27, 2023, to identify empirical studies that conducted a quantitative analysis on the trends in child marriage (ie, aged <18 years for girls and <21 years for boys) in the states of India using nationally representative data for at least two time periods. We used structured combinations of keywords (“Child Marriage” OR “Early Marriage” OR “Adolescent Marriage” OR “Teenage Marriage” OR “Teen Marriage” OR “Forced Marriage” OR “Child Bride” OR “Child Groom OR “Age at first marriage”) AND “India” AND “states” AND (“Trends” OR “Change”), without any constraints on year of publication and only in English. The search yielded 463 studies, of which we shortlisted 31 studies after screening the titles and abstracts. After duplicates were deleted, 15 articles were shortlisted. Of these 15 studies, seven studies provided state-level estimates for at least one time period, and six studies provided national or sub-national estimates of child marriage or age at first marriage for at least two time periods. Further, ten studies provided estimates of child marriage or age at first marriage using the National Family Health Surveys, of which only two studies provided estimates for child and early marriages across states of India for at least two time periods. Two studies provided the prevalence of child marriage and its rate of change at the all-India levels between 1993 and 2016. The first study estimated only mean age at marriage across the states between 1993 and 2016, whereas the second study explored early marriage in India between 2005 and 2016 across all states and Union Territories of India. However, it provided rate of change only at national levels. Another study explored age at first marriage in India between 1993 and 2021 and provided the percentage of women who married at exact ages in single years including adolescent ages.

**Added value of this study**

Our study has provided, to our knowledge, the first comprehensive assessment of the prevalence of child marriage across all states and Union Territories of India between 1993 and 2021 and its changes. Given changes in the geographical boundaries of states and Union Territories during the study period, we align these over time to correspond to their current geography from 2021. Further, we estimated the total number (headcount) of girls and boys between the ages of 20–24 years who were married before the age of 18 years**.** For girls we estimated the headcounts across states for 1993 and 2021 and for boys we estimated the headcounts across states for 2006 and 2021. We observed that in the past 30 years, the prevalence of child marriage in girls has declined from 49·4% in 1993 to 22·2% in 2021. Child marriage in boys declined from 7·1% (95% CI 6·9–30·8) in 2006 to 2·2% (1·8–2·7) in 2021. Although child marriage in girls and boys has declined at the national level from 1993 to 2021, the subnational variation suggests possible stagnation in the rate of decline. Further, the headcount burden has indicated that the absolute number of boys and girls subjected to child marriage increased in several states over time, despite decreasing prevalence. These results have important implications for how success in meeting Sustainable Development Goal 5.3 is tracked in India.

**Implications of all the available evidence**

Considering the trends in child marriage and its current headcount, policy frameworks that cater to prohibition and control of child marriages should be carefully reviewed for their implementation challenges. States such as Bihar, Uttar Pradesh, West Bengal, and Rajasthan that currently witness a high burden and prevalence of child marriage should be given focused attention. Child marriages have long-term implications on the fertility, health, and mortality patterns that adversely affect the economy and population well-being. Despite stringent laws that penalise child marriages, one in five girls and nearly one in six boys in India are still married below the legal age of marriage, which is worrisome and reflects the inadequacy of current policy designs.

We further estimate the population headcount burden of child marriage in girls and boys for all of India, and each of the states and Union Territories in 1993 for girls, 2006 for boys, and 2021 for both.

## Methods

### Study design

For this cross-sectional study, we used all five waves of the National Family Health Survey (NFHS) conducted in 1992–93 (NFHS-1), 1998–1999 (NFHS-2), 2005–06 (NFHS-3), 2015–16 (NFHS-4), and 2019–21 (NFHS-5).^9^, ^10^, ^11^, ^12^ The survey uses a multistage stratified cluster sampling design that has been documented elsewhere.^11^ Population data were obtained from the Census of India in 1991, 2001, and the most recent population projections published for 2021.^13^, ^14^

The NFHS surveys underwent ethical approval by the ICF Institutional Review Board and the International Institute for Population Sciences Institutional Review Board, and were reviewed by the US Centers for Disease Control and Prevention.^9^, ^11^ The Harvard Longwood Institutional Review Board came to the conclusion that this study did not meet the regulatory definition of research with human subjects and was exempt from ethical review.

### Participants

Women and men aged 20–24 years were included in the study population to align with the SDG indicator 5.3.1. In the 1993 and 1999 waves, only ever-married women were eligible to participate in the full women's survey module in which age at first marriage was collected. Data on unmarried women were obtained from the household member listing (appendix p 2). Both married and unmarried women were eligible for the full women's survey module in 2006, 2016, and 2021. Men's age at first marriage was unavailable in 1993 and 1998 as the survey did not collect this information in these years. The final analytic samples for women and men are presented in the appendix (p 14).

### Definition of child marriage

We defined child marriage as women and men aged 20–24 years married before their 18th birthday to align with the definition of the SDG target 5.3.1. We note that the legal age of marriage in India for men is 21 years and the legal age for women in India is 18 years. Respondents were asked the month and year in which they began their first cohabitation and their age at first marriage. No missing values on age at first marriage were included in the published dataset, which were handled according to the standardised, published methodology of the Demographic and Health Survey (NFHS is the name given to the Demographic and Health Surveys implemented in India).^15^ The age of unmarried women in NFHS-1 and NFHS-2 was obtained from the head of the household.

### Statistical analysis

As the geographical boundaries of states and Union Territories have evolved over time, we constructed comparable state estimates in each survey wave. In 1993, there were 25 states and seven Union Territories in India. As a result of changes in administrative boundaries and the creation of new states, India had 28 states and eight Union Territories as of 2021. We assigned districts surveyed in earlier survey years to their current states and Union Territories following a process described elsewhere.^16^

We first calculated the prevalence of child marriage over time for all-India and states and Union Territories at each survey point, accounting for the multistage stratified cluster sampling design using survey weights. We then calculated the standardised absolute change to quantify the change (in percentage points) in the prevalence of child marriage across time periods: *AC*=*P*_t_ – *P*_x_ where *P*_t_ refers to the prevalence in the most recent year and *P*_x_ refers to the prevalence in a previous year. Given the variation in years between surveys, we standardised absolute change by dividing the absolute change by the number of years between each survey. Thus, a negative standardised absolute change indicates a decline in the prevalence of child marriage, whereas a positive value indicates an increase in prevalence.

We used visual and descriptive methods to assess state-level patterns over time. Box plots were used to assess whether state level inequalities had increased or decreased over time. We assessed the magnitude of change in child marriage at state-level from 1993 to 2021 for girls and from 2006 to 2021 for boys associated with the baseline state prevalence of child marriage with simple linear regression. Headcount burden refers to the number of child marriages for boys and girls that took place in each time period, weighted for survey design (appendix p 3). We estimated the headcount burden of child marriage using the methods provided by the Integrated Public Use Microdata Series^17^ and examined the correlation between state-level prevalence and headcount burden for child marriage. We further estimated the headcount burden in 1993 for girls and 2006 for boys and compared both with the headcount burden in 2021 for all-India and for states and Union Territories by comparing the standardised absolute change in prevalence with the percentage change in headcount between 1993 (for girls) and 2006 (for boys) with 2021. We used Stata SE (version 15.0) for the analysis and R (version 4.2.2) for graphics. We have reported our study according to the STROBE statement for observational studies (appendix pp 4–5).

### Role of the funding source

The funder of the study had no role in study design, data collection, data analysis, data interpretation, or writing of the report.

## Results

A description of the sample across demographic and socioeconomic characteristics in each survey wave can be found in the appendix (pp 15–16). An interactive view of the state maps and data is available via a dashboard.

**Figure 1:**
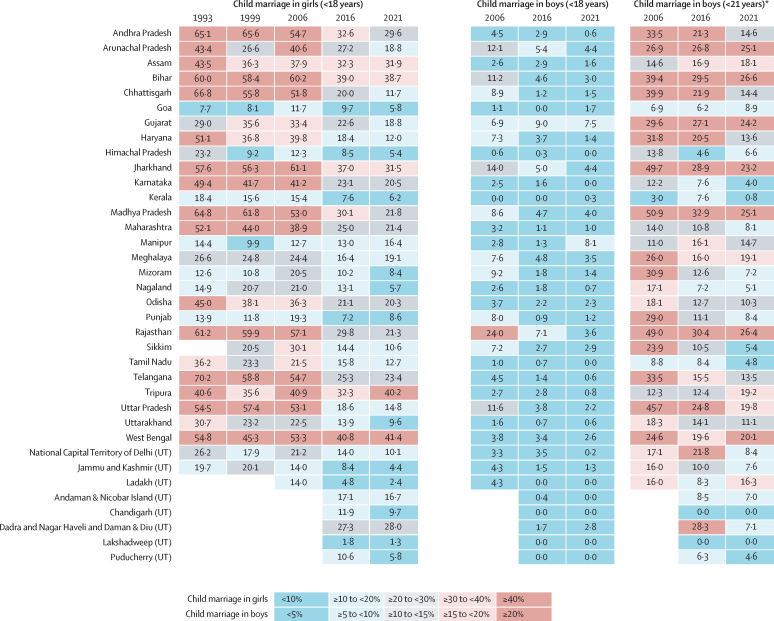
All-India prevalence of child marriage, 1993–2021 Prevalence estimates of child marriage in girls and boys for each state and Union Territory at each available year. No estimates are provided for some states for years where no data are available. UT=Union Territory. * We provide estimates for boys married younger than 21 years to correspond with India's child marriage law, which defines child marriage in boys to be younger than 21 years.

Child marriage declined considerably during the study period (1993–2021 for girls and 2006–2021 for boys). The all-India prevalence of child marriage in girls declined from 49·4% (95% CI 48·1–50·8) in 1993 to 22·3% (21·9–22·7) in 2021 (figure 1), and the all-India prevalence of child marriage in boys declined from 7·1% (6·9–30·8) in 2006 to 2·2% (1·8–2·7) in 2021. Substantial variation exists in the change in prevalence of girl and boy child marriage across the states and Union Territories during the study period (appendix pp 17–20). Estimates using the Indian legal definition of boy child marriage (married before their 21st birthday) can be found in the appendix (pp 21–23).

**Figure 2:**
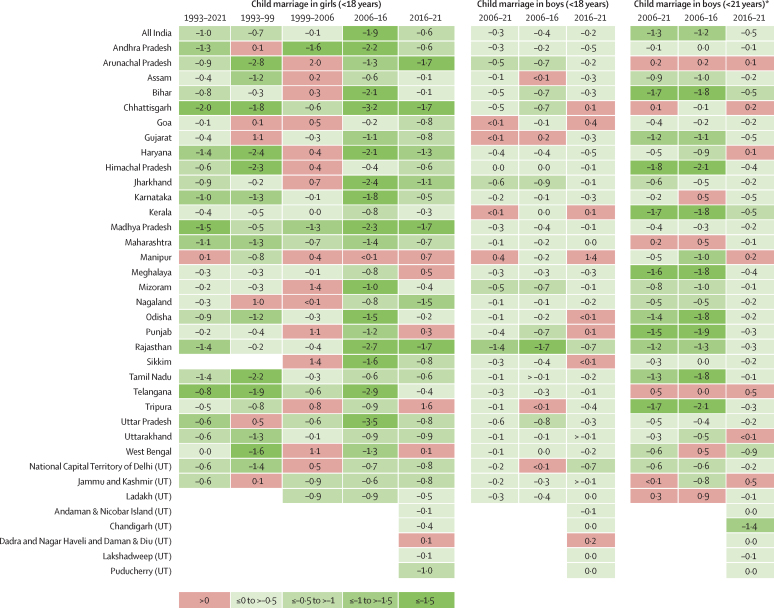
Standardised absolute change in prevalence of child marriage by state and Union Territory, 1993–2021 Standardised absolute change in prevalence of child marriage in girls and boys for each state and Union Territory at each available year (most recent wave to previous wave). A positive value denotes increase in prevalence. No estimates are provided for some states for years in which no data are available. UT=Union Territory. * We provide estimates for boys married younger than 21 years to correspond with India's child marriage law, which defines child marriage in boys to be younger than 21 years.

All states, except Manipur, experienced a decline in the prevalence of girl child marriage between 1993 and 2021 (figure 2; appendix pp 23–24). Between 1993 and 1999, 20% (six of 30) of states and Union Territories experienced increases in girl child marriage (standardised absolute change>0), whereas between 1999 and 2006, 50% (15 of 30) of states and Union Territories experienced increases in girl child marriage (standardised absolute change>0). The period between 2006 and 2016 was characterised by accelerated reductions in child marriage in girls. Manipur was the only state and Union Territory that experienced an increase in the prevalence of child marriage in girls; however, this increase was far smaller than the previous increase Manipur had between 1999 and 2006. Between 2019 and 2021, the magnitude of reduction in child marriage in girls across states and Union Territories was smaller than the reduction that occurred between 2006 and 2016. During the period between 2016 and 2021, the standardised absolute mean change prevalence across all states was –0·6 percentage points, which was the lowest of any time period examined. Six (16·7%) of 36 states or Union Territories saw an increase in prevalence in child marriage in girls during 2016–2021, with the increases observed in Manipur and Tripura being greater than during any previous period. 25 states and Union Territories saw a deceleration in the 2016–21 standardised absolute change compared with 2006–16.

For child marriage in boys, the standardised absolute change between 2006 and 2021 across states and Union Territories ranged from –1·3 in Rajasthan to 0·3 in Manipur. Four states and Union Territories experienced overall increases in the prevalence of child marriage in boys from 2006 to 2021. As with child marriage in girls, the reductions in prevalence were greater between 2006 and 2016 compared with between 2016 and 2021. Only three states and Union Territories saw increases in child marriage in boys between 2006 and 2016 compared with eight states and Union Territories between 2016 and 2021. Of the remaining 25 states and Union Territories with available data, 48% (12 of 25) experienced a slower rate of reduction in child marriage in boys between 2016 and 2021 compared with 2006–16.

**Figure 3:**
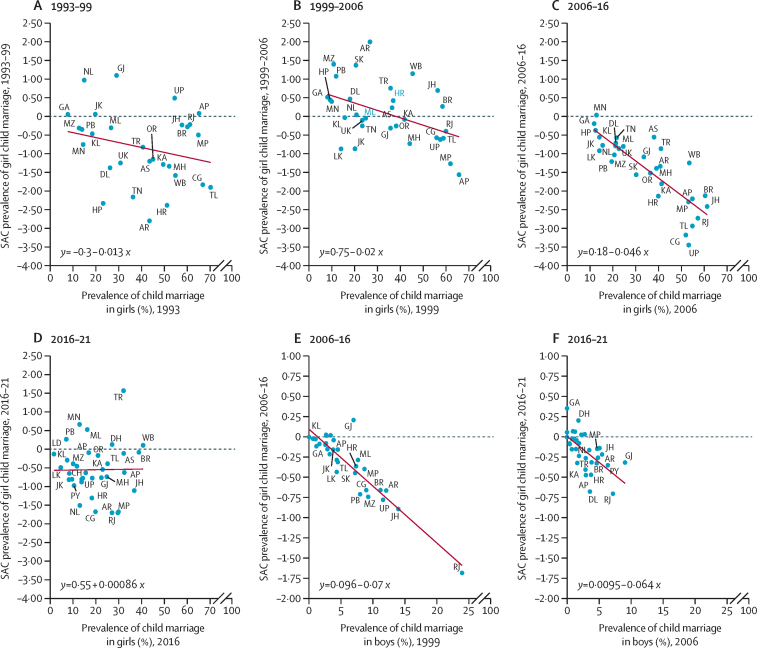
Association between baseline prevalence and standardised absolute change in prevalence in child marriage, 1993–2021 Graphs show association between baseline prevalence and standardised absolute change in prevalence in child marriage for the periods (A) 1993–99, (B) 1999–2006, (C) 2006–16, (D) 2016–21, (E) 2006–16, and (F) 2016–21. AP=Andhra Pradesh. AR=Arunachal Pradesh. AS=Assam. BR=Bihar. CG=Chhattisgarh. CH=Chandigarh. DH=Dadra and Nagar Haveli and Daman and Diu. DL=National Capital Territory of Delhi. GA=Goa. GJ=Gujarat. HP=Himachal Pradesh. HR=Haryana. JH=Jharkhand. JK=Jammu & Kashmir. KA=Karnataka. KL=Kerala. LD=Lakshadweep. LK=Ladakh. MH=Maharashtra. ML=Meghalaya. MN=Manipur. MP=Madhya Pradesh. MZ=Mizoram. NL=Nagaland. OR=Odisha. PB=Punjab. PY=Puducherry. RJ=Rajasthan. SAC=standardised absolute change. SK=Sikkim. TL=Telangana. TN=Tamil Nadu. TR=Tripura. UK=Uttarakhand. UP=Uttar Pradesh. WB=West Bengal.

Overall, there was an inverse relationship between the state and Union Territory prevalence of child marriage and the standardised absolute change in such prevalence from 1993 for girls and 2006 for boys (appendix p 9). Figure 3 shows that for child marriage in girls, there was an inverse association between baseline prevalence and standardised absolute change in prevalence during the periods before 2016, with the strongest negative association occurring between 2006 and 2016, suggesting that states with a higher prevalence of child marriage declined more rapidly in this period. Conversely, during the most recent period (2016–21), no association between baseline prevalence and standardised absolute change in prevalence was found. For child marriage in boys, the negative association between baseline prevalence and standardised absolute change in prevalence was similar between 2006 and 2016, and 2016 and 2021.

**Figure 4:**
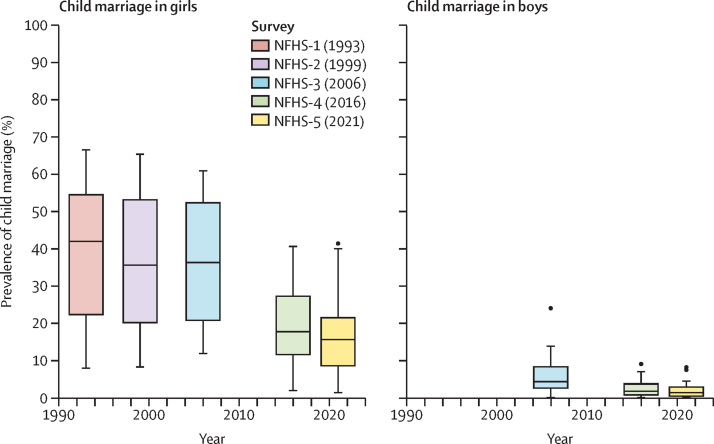
Summary distribution of child marriage across states and Union Territories of India, 1993–2021 The median is represented by a dark line inside each rectangle. The IQR is shown by the length of the rectangle. The extent of the whiskers shows data that are 1·5 times the IQR. Outliers are shown outside the extent of the whiskers when they are 1·5 times greater or smaller than the IQR. NFHS= National Family Health Survey.

Inequality across states and Union Territories in the prevalence of child marriage reduced over time (figure 4). The IQR for the prevalence of child marriage in girls decreased from 31·6% (23·2–54·8%) to 12·9% (8·6–21·5%) in 2021. Minimal reductions in state and Union Territory inequality in the prevalence of child marriage in girls occurred between 1993 and 2006. The largest reductions in inequality occurred between 2006 and 2016. Reductions in state and Union Territory inequality in the prevalence of child marriage in boys were observed between 2006 and 2021. The largest reduction occurred between 2006 and 2016. In 2006, the IQR for the prevalence of child marriage in boys was 18·3% (14·4–32·7%). In 2021, the IQR decreased to 11·6% (7·8–19·5%), which was approximately the same as in 2016. In 2006, there was nearly double the state and Union Territory inequality in child marriage in girls than in boys. In 2016, the IQR was only slightly larger across states for child marriage in girls than boys, and by 2021, the difference in the IQR for child marriage in girls versus boys across states and Union Territories was minimal.

The Spearman rank correlation between the rank order of states in 1993 and 2021 for child marriage in girls suggests a strong relationship (*r*=0·70; p<0·001), meaning the rank order of states did not change much between 1993 and 2021. States and Union Territories that had the highest prevalence of child marriage in girls in 1993 tended to be among the states with the highest prevalence in 2021 (appendix p 11). A few notable exceptions exist; the rank of Chhattisgarh fell from number 2 in 1993 to number 22 in 2021, and Uttar Pradesh fell from number 9 in 1993 to number 19 in 2021. West Bengal's rank increased from number 8 to number 2, and Tripura's rank increased from number 16 to number 2. The Spearman rank correlation between the rank order of states in 2006 and 2021 for child marriage in boys also suggests a strong relationship (*r*=0·61; p<0·001). Several exceptions also were observed; Manipur and Gujarat were notable for their increase in rank between 2006 and 2021.

We estimated that in 2021, the headcount of child marriage in girls was 13 464 450 and 1 454 894 in boys (table 1). Four states (Bihar [16·7%], West Bengal [15·2%], Uttar Pradesh [12·5%], and Maharashtra [8·2%]) accounted for more than half of the total headcount burden of child marriages in girls. For boys, Gujarat (29·0%), Bihar (16·5%), West Bengal (12·9%), and Uttar Pradesh (8·2%) accounted for more than 60% of the headcount burden. For both girls and boys, states with a higher prevalence tended to also have a high headcount burden (appendix pp 12–13). For child marriage in both girls and boys in 2021, Uttar Pradesh was a notable outlier for having a relatively low prevalence but high burden, and Tripura was an outlier with a high prevalence but low burden. For child marriage in boys, Maharashtra was an outlier for having a high burden but low prevalence, whereas Manipur has a high prevalence but fairly low burden.

**Table 1:** Estimated headcount of the number of child marriages in girls and boys for India and 36 states and Union Territories, and percentage share of child marriage of each state and Union Territory to all-India, 2021

	**Child marriages in girls (N=13 464 450)**			**Child marriages in boys (N=1 454 894)**	
	Estimated headcount (n)	%	States and Union Territories	Estimated headcount (n)	%
Bihar	2 244 631	16·7%	Gujarat	422 007	29·0%
West Bengal	2 049 788	15·2%	Bihar	240 136	16·5%
Uttar Pradesh	1 680 312	12·5%	West Bengal	188 036	12·9%
Maharashtra	1 108 023	8·2%	Uttar Pradesh	119 451	8·2%
Rajasthan	889 958	6·6%	Maharashtra	114 709	7·9%
Madhya Pradesh	809 570	6·0%	Rajasthan	79 711	5·5%
Andhra Pradesh	602 123	4·5%	Madhya Pradesh	77 607	5·3%
Karnataka	564 940	4·2%	Assam	39 555	2·7%
Jharkhand	539 160	4·0%	Jharkhand	28 909	2·0%
Gujarat	513 619	3·8%	Odisha	20 799	1·4%
Assam	509 557	3·8%	Andhra Pradesh	19 879	1·4%
Odisha	407 001	3·0%	Telangana	16 481	1·1%
Tamil Nadu	386 543	2·9%	Chhattisgarh	13 477	0·9%
Telangana	339 451	2·5%	Manipur	13 132	0·9%
Chhattisgarh	180 752	1·3%	Jammu and Kashmir	12 187	0·8%
Haryana	138 806	1·0%	Punjab	11 229	0·8%
Punjab	96 129	1·0%	Haryana	10 685	0·7%
National Capital Territory of Delhi	93 600	1·0%	Meghalaya	7619	1·0%
Kerala	74 831	1·0%	Kerala	6424	0·4%
Tripura	61 776	1·0%	Goa	2824	<1·0%
Uttarakhand	51 884	<1·0%	Tripura	1890	<1·0%
Meghalaya	28 625	<1·0%	Uttarakhand	1754	<1·0%
Jammu and Kashmir	25 513	<1·0%	National Capital Territory of Delhi	1333	<1·0%
Manipur	15 254	<1·0%	Dadra and Nagar Haveli	1268	<1·0%
Himachal Pradesh	14 306	<1·0%	Mizoram	1022	<1·0%
Arunachal Pradesh	8782	<1·0%	Arunachal Pradesh	1017	<1·0%
Dadra and Nagar Haveli	6569	<1·0%	Sikkim	990	<1·0%
Goa	4243	<1·0%	Nagaland	763	<1·0%
Chandigarh	3910	<1·0%	Andaman and Nicobar Island	0	<1·0%
Nagaland	3603	<1·0%	Chandigarh	0	<1·0%
Mizoram	3315	<1·0%	Himachal Pradesh	0	<1·0%
Sikkim	2711	<1·0%	Karnataka	0	<1·0%
Puducherry	2646	<1·0%	Ladakh	0	<1·0%
Andaman and Nicobar Island	2270	<1·0%	Lakshadweep	0	<1·0%
Ladakh	214	<1·0%	Puducherry	0	<1·0%
Lakshadweep	35	<1·0%	Tamil Nadu	0	<1·0%

States and Union Territories are ordered from the highest to lowest percentage share of child marriage.

The overall headcount of child marriage in girls decreased by just more than 5 million individuals between 1993 and 2021, although seven states saw an increase in headcount during this period (table 2). The largest absolute increase in headcount was observed in West Bengal, representing an increase of 32·3% (difference n=500 346 individuals girls) in headcount. Jharkhand had the largest percentage increase in headcount (53·1% [difference n=186 936]) between 1993 and 2021. Assam and Bihar also saw increases in which more than 50 000 more women married early in 2021 (representing an increase of 13·1%) than in 1993 (representing an increase of 2·6%).

**Table 2:** Estimated headcount of the number of child marriages in girls and boys for India and 36 states and Union Territories, absolute difference in headcount, and percentage change in headcount, 1993–2021

		**Headcount 1993**	**Headcount 2021**	**Difference 1993–2021**	**Percent change in headcount 1993–2021**	**All-India, states, and Union Territories**	**Headcount 2006**	**Headcount 2021**	**Difference 2006–21**	**Percent change in headcount 2006–21**
India		18 484 074*	13 464 450	−5 019 624	−27·2%	India	3 738 821	1 454 894	−2 283 927	−61·1%
States and Union Territories										
	Jharkhand	352 224	539 160	186 936	53·1%	Manipur	3236	13 132	9896	305·8%
	West Bengal	1 549 442	2 049 788	500 346	32·3%	Goa	898	2824	1926	214·5%
	Tripura	48 789	61 776	12 987	26·6%	Gujarat	190 165	422 007	231 842	121·9%
	Goa	3744	4243	499	13·3%	West Bengal	174 391	188 036	13 645	7·8%
	Assam	450 447	509 557	59 110	13·1%	Assam	36 906	39 555	2649	7·2%
	Manipur	13 583	15 254	1671	12·3%	Bihar	325 592	240 136	−85 456	−26·3%
	Bihar	2 188 716	2 244 631	55 915	2·6%	Meghalaya	12 249	7619	−4630	−37·8%
	Gujarat	539 097	513 619	−25 478	−4·7%	Jammu and Kashmir	22 540	12 187	−10 353	−45·9%
	National Capital Territory of Delhi	104 850	93 600	−11 250	−10·7%	Maharashtra	214 064	114 709	−99 355	−46·4%
	Rajasthan	1 019 320	889 958	−129 362	−12·7%	Sikkim	2540	990	−1550	−61·0%
	Meghalaya	33 770	28 625	−5145	−15·2%	Nagaland	2006	763	−1243	−62·0%
	Punjab	117 370	96 129	−21 241	−18·1%	Uttarakhand	5376	1754	−3622	−67·4%
	Mizoram	4290	3315	−975	−22·7%	Odisha	64 792	20 799	−43 993	−67·9%
	Odisha	624 315	407 001	−217 314	−34·8%	Tripura	6201	1890	−4311	−69·5%
	Maharashtra	1 831 524	1 108 023	−723 501	−40·0%	Madhya Pradesh	271 246	77 607	−193 639	−71·4%
	Madhya Pradesh	1 352 986	809 570	−543 416	−40·2%	Mizoram	4374	1022	−3352	−76·6%
	Andhra Pradesh	1 019 920	602 123	−417 797	−41·0%	Jharkhand	166 465	28 909	−137 556	−82·6%
	Karnataka	1 028 214	564 940	−463 274	−45·0%	Arunachal Pradesh	6648	1017	−5631	−84·7%
	Arunachal Pradesh	16 895	8782	−8113	−48·0%	Uttar Pradesh	931 953	119 451	−812 502	−87·2%
	Uttarakhand	101 505	51 884	−49 621	−48·9%	Rajasthan	667 330	79 711	−587 619	−88·1%
	Tamil Nadu	767 286	386 543	−380 743	−49·6%	Andhra Pradesh	172 107	19 879	−152 228	−88·5%
	Uttar Pradesh	3 350 584	1 680 312	−1 670 272	−49·9%	Haryana	95 642	10 685	−84 957	−88·8%
	Telangana	740 784	339 451	−401 333	−54·2%	Punjab	125 442	11 229	−114 213	−91·1%
	Haryana	367 172	138 806	−228 366	−62·2%	National Capital Territory of Delhi	33 406	1333	−32 073	−96·0%
	Chhattisgarh	543 077	180 752	−362 325	−66·7%	Chandigarh	90 825	0	−90 825	−100·0%
	Nagaland	11 055	3603	−7452	−67·4%	Himachal Pradesh	1422	0	−1422	−100·0%
	Kerala	248 864	74 831	−174 033	−69·9%	Karnataka	84 213	0	−84 213	−100·0%
	Himachal Pradesh	54 251	14 306	−39 945	−73·6%	Tamil Nadu	26 792	0	−26 792	−100·0%
	Jammu and Kashmir	..	25 513	..	..	Ladakh	22 540	0	−22 540	−100·0%
	Dadra and Nagar Haveli	..	6569	..	..	Kerala	0	6424	6424	..
	Chandigarh	..	3910	..	..	Telangana	..	16 481	..	..
	Sikkim	..	2711	..	..	Chhattisgarh	..	13 477	..	..
	Puducherry	..	2646	..	..	Dadra and Nagar Haveli	..	1268	..	..
	Andaman and Nicobar Island	..	2270	..	..	Andaman and Nicobar Island	..	0	..	..
	Ladakh	..	214	..	..	Lakshadweep	..	0	..	..
	Lakshadweep	..	35	..	..	Puducherry	..	0	..	..

Difference was calculated as the headcount in 2021 minus the headcount in 1993 for child marriages in girls and in 2006 for child marriages in boys. A positive number reflects an increase in headcount and a negative number reflects a decrease in headcount. States and Union Territories are ordered according to percentage change in headcount 1993–2021 (highest to lowest)**.**

* Excludes Jammu and Kashmir.

Most states and Union Territories saw a decrease in headcount of child marriage in girls between 1993 and 2021. Uttar Pradesh had the most substantial absolute decrease (difference n=1 670 272), which accounted for an estimated one-third of the all-India decrease in headcount of child marriage in girls observed between 1993 and 2021. West Bengal saw the largest absolute increase with over 500 000 more girls married as children (table 2).

Although smaller in the absolute number compared with girls, Manipur, Goa, and Gujarat saw substantial increases in the magnitude of the child marriage in boys headcount between 2006 and 2021. In Gujarat, the headcount burden of child marriage in boys increased by 121·9% (difference n=231 842; table 2). Headcount estimates by state following the Indian legal definition of child marriage in boys are available in the appendix (p 25).

## Discussion

Our study has three substantial findings. First, although there have been dramatic declines in child marriage during the last three decades, there is evidence of stagnation. The largest reductions in child marriage occurred between 2006 and 2016. Both the slowed rate of decline and increases observed in prevalence at the state and Union Territory level between 2016 and 2021 could suggest a stall. Second, there have been improvements in state-level inequalities in the prevalence of child marriage. These improvements were most pronounced between 2006 and 2016, with little improvement after 2016. Third, using both prevalence and total headcount as metrics in concert could better identify the states and Union Territories where intervention is urgently needed. Although in general, states and Union Territories with the highest prevalence of child marriage in 2021 accounted for the largest headcount, there were important exceptions in which identifying priority states and Union Territories on prevalence alone might not have the greatest effect on the absolute number affected by the practice. Change in the headcount burden of child marriage in girls over time raises concerns about over-reliance on prevalence as the key metric as several states experienced substantial increases in headcount, despite marked decreases in prevalence during the last three decades. In the context of high population growth, relying only on prevalence alone as an indicator of progress could overstate success towards achieving the SDG target 5.3.

In many countries, increased global attention to ending child marriage has been coupled with the passing of legislation to ban child marriage during the last decade. Advocacy efforts continue to focus on closing the legal loopholes that continue to allow the practice.^18^ Although some research points to the positive effect of minimum marriage age laws on reducing the prevalence of child marriage in countries that have implemented them,^19^ others argue that such laws are difficult to enforce, especially in rural and hard-to-reach areas or in settings where other authorities, including religious institutions, can grant marriages outside of government oversight,^20^ ultimately rendering little effect of the laws.^21^ Further, child marriage bans could be accompanied by poor enforcement, thereby limiting their effect.^22^

Policy response to prevent child marriage in India has involved legal prohibition intended to directly prevent child marriage, whereas other policies and schemes have been implemented to address its underlying drivers, with such programmes focusing on expanding social protections, increasing girls’ education, and reducing economic vulnerability (appendix pp 27–31). The Prohibition of Child Marriage Act passed in 2006 sets the legal age of marriage for men to 21 years and women to 18 years. There was considerable publicity of the Prohibition of Child Marriage Act at the time of its passing, and notably, it increased the punishment for child marriage to up to 2 years’ imprisonment and requested a substantial fine for individuals involved in the marriage.^23^ Of note, the period between 2006 and 2016 saw the largest reductions in child marriage in India during the last three decades. Current legislation is under debate that would raise the legal age of marriage for women to 21 years.^24^ Individual states, such as Uttar Pradesh, have also introduced legislation intended to strengthen laws that prohibit the practice of child marriage. State-level governments have been crucial partners to strengthen efforts to end child marriage.

Some states stand out as positive deviants. Uttar Pradesh is an example of a state that has achieved dramatic decreases in prevalence and headcount for child marriage in girls, yet, other states have struggled, such as West Bengal. The scope of our study does not permit a detailed examination of policy and intervention at the state level that could influence the changing landscape of child marriage over time. Decisions made to implement such programmes could be driven by political actors operating at local levels, such as districts, thus state-level estimates could mask variation among lower-level administrative units. More research is needed to understand district-level variation and drivers of child marriage.

To our knowledge, this study is the first to provide robust sub-national estimates of changes in prevalence over time and headcount of child marriage in India using a methodology to make state-level and Union Territory-level estimates comparable over time. Other research has estimated trends over time for select states by using birth cohorts in NFHS-4 (2015–16) then performing adjustments to account for under-reporting.^25^ The report suggests a higher prevalence of child marriage in girls in the early to mid-1990s than we have estimated. Such differences could be due to the adjustment procedure, or the results of survival bias among the older age cohorts. For 1993 and 1999, our all-India estimates for child marriage in girls are largely consistent with what was reported in the official NFHS publications for the age cohort of women aged 20–24 years.

The results of this study are subject to some limitations. First, as the ages of unmarried women were reported by the head of the household in the NFHS-1 and NFHS-2, it is possible that such characteristics were misreported, resulting in misclassification. Such misclassification of the ages of unmarried women is assumed to be at random, and we would not expect bias to result. We examined this assumption by calculating the correlation between the age reported by the head of the household and the self-reported age for women in 5-year categories across all ages. In both surveys, the correlation was greater than 0·95, suggesting limited misclassification. Second, geographical coverage of the NFHS has expanded over time, with more recent surveys from 2016 and 2021 offering representative samples at the district level. Despite the smaller sample size in 1993 and 1998, we do not believe that it would affect generalisability at the state and Union Territory level given the random sampling scheme. Finally, NFHS-5 was conducted during the COVID-19 pandemic. An examination of survey interview reveals that 70% of the survey was complete before March, 2020, and less than 4% remained at the time of India's second COVID-19 wave in April, 2021. The procedures undertaken to ensure safety and data quality during the pandemic are described elsewhere.^11^

Warnings were issued about the increased risk of child marriage brought by the pandemic, with some estimates suggesting that the pandemic could result in more than half a million excess child marriages.^26^ As our study only included participants who were no longer at risk of incident child marriage due to their age, our results do not reflect these increases. The stagnation that we observe could be amplified across future survey waves if pandemic-related increases in the practice occurred.

Several sources of bias and error could have affected our results, including social desirability bias, recall bias, changes in cultural understanding of the definition of marriage over time, and errors in survey administration.^27^ Women have been found to change their reported age of marriage based on social expectations. In India, changes in the social acceptability and legality of child marriage could have led to it being over-reported during the earlier NFHS waves and under-reported during later waves. Such changes might have had an effect on the reductions observed after 2006 following the widespread publicity of the Prohibition of Child Marriage Act. Limiting the study population to participants aged 20–24 years partially is partially due to on research suggesting that women aged 20–24 years could be more likely to report early age of marriage than younger women.^28^ Data on marriage in the Demographic and Health Surveys (of which the NFHS is part), including surveys conducted in the 1980s and 1990s, are considered to be of high quality. No evidence was found in the NFHS-1 and NFHS-2 to suggest improper identification of eligible women based on age and marital status within households; age was missing for less than 0·01% of people listed on the household schedule in the NFHS-1 and NFHS-2.^29^ Across all NFHS waves, fewer than 0·5% of respondents were missing their age at first marriage. When age of marriage is incomplete in a Demographic and Health Survey, it is typically only the month that is missing or inconsistent.^30^

Countries globally struggle with the best way to eliminate child marriage and continue to pledge action.^31^ In India, changes to national-level and state-level legislation related to the legal age of marriage for both boys and girls remains the subject of active debate. Child marriage is not just a concern in low-income and middle-income countries; in fact, marriage before the age of 18 years is legal in the majority of US states, and an estimated 300 000 children were married in the USA between 2000 and 2018.^32^ There remain important evidence gaps about how to most effectively eliminate child marriage in diverse settings globally; however, some interventions, such as cash transfers, have shown poor success in certain geographies, such as India.^5^ The possible state-level and Union Territory-level stagnation observed in India in reducing child marriage during the last several decades is a big concern. Re-igniting progress in states and Union Territories with the highest prevalence and burden of child marriage in India is necessary to achieve the SDG target 5.3.

## Equitable partnership declaration

## Data sharing

Underlying data can be accessed from https://dhsprogram.com/data/available-datasets.cfm.

## Declaration of interests

We declare no competing interests.

## Supplementary Materials

Supplementary appendix 1

Equitable Partnership Declaration

## References

[1] Fan S, Koski A (2022). The health consequences of child marriage: a systematic review of the evidence. BMC Public Health.

[2] Wodon Q, Male C, Nayihouba A (2017). Economic impacts of child marriage: global synthesis report. https://documents1.worldbank.org/curated/en/530891498511398503/pdf/116829-WP-P151842-PUBLIC-EICM-Global-Conference-Edition-June-27.pdf.

[3] UNICEF (2018). Child Marriage: latest trends and future prospects. https://data.unicef.org/resources/child-marriage-latest-trends-and-future-prospects.

[4] Gastón CM, Misunas C, Cappa C (2019). Child marriage among boys: a global overview of available data. Vulnerable Child Youth Stud.

[5] Malhotra A, Elnakib S (2021). 20 years of the evidence base on what works to prevent child marriage: a systematic review. J Adolesc Health.

[6] Plesons M, Travers E, Malhotra A (2021). Updated research gaps on ending child marriage and supporting married girls for 2020-2030. Reprod Health.

[7] Cappa C, Murray C, Maksud N (2023). Child marriage could be history by 2030, or last 300 more years. Lancet.

[8] McDougal L, Shakya H, Dehingia N (2020). Mapping the patchwork: exploring the subnational heterogeneity of child marriage in India. SSM Popul Health.

[9] International Institute for Population Sciences (IIPS) and ICF (2017).

[10] International Institute for Population Sciences (2007).

[11] International Institute for Population Sciences and ICF (2021).

[12] Roy T (2000).

[13] Office of the Registrar General & Census Commissioner, India (1994).

[14] Office of the Registrar General & Census Commissioner (1993).

[15] Croft T (1991).

[16] Subramanian S, Ambade M, Sharma S, Kumar A, Kim R (2023). Prevalence of Zero-Food among infants and young children in India: patterns of change across the States and Union Territories of India, 1993–2021. EClinicalMedicine.

[17] IPUMS Demographic and Health Surverys (2023). Health-related microdata for low- and middle-income countries. https://www.idhsdata.org/idhs/index.shtml.

[18] Gausman J, Othman A, Amawi A, Langer A (2019). Child marriage in the Arab world. Lancet.

[19] Maswikwa B, Richter L, Kaufman J, Nandi A (2015). Minimum marriage age laws and the prevalence of child marriage and adolescent birth: evidence from sub-Saharan Africa. Int Perspect Sex Reprod Health.

[20] Wodon QT, Tavares PMT, Fiala O, Nestour AL, Wise L (2017).

[21] Batyra E, Pesando LM (2021). Trends in child marriage and new evidence on the selective impact of changes in age-at-marriage laws on early marriage. SSM Popul Health.

[22] Collin M, Talbot T (2023). Are age-of-marriage laws enforced? Evidence from developing countries. J Dev Econ.

[23] Tambe A (2019).

[24] PRS Legislative Research (2021). The prohibition of child marriage (amendment) bill, 2021. https://prsindia.org/billtrack/the-prohibition-of-child-marriage-amendment-bill-2021.

[25] Fund UNCs (2019).

[26] Yukich J, Worges M, Gage AJ (2021). Projecting the impact of the COVID-19 pandemic on child marriage. J Adolesc Health.

[27] Liang M, Simelane S, Chalasani S, Snow R (2021). New estimations of child marriage: evidence from 98 low- and middle-income countries. PLoS One.

[28] Neal SE, Hosegood V (2015). How reliable are reports of early adolescent reproductive and sexual health events in demographic and health surveys?. Int Perspect Sex Reprod Health.

[29] International Institute for Population Sciences (1995).

[30] Pullum TW, Staveteig S (2017).

[31] Indira FN, Luseba GN, Tesfaye E, Tallen PK (2023). Towards a world with no child marriage: four countries pledge action. Lancet.

[32] Reiss F (2021). Child marriage in the United States: prevalence and implications. J Adolesc Health.

