# Supplementary material for: Prevalence of girl and boy child marriage across states and Union Territories in India, 1993–2021: a repeated cross-sectional study
Source: Lancet Glob Health. 2023 Dec 15;12(2):e271–81. doi: 10.1016/S2214-109X(23)00470-9 (PMC10805006; doi:10.1016/S2214-109X(23)00470-9)
Supplement: Supplementary appendix 1 [file mmc1.pdf]

THE LANCET
Global Health

Supplementary appendix 1

This appendix formed part of the original submission and has been peer reviewed. We post it as supplied by the authors.

Supplement to: Gausman J, Kim R, Kumar A, Ravi S, Subramanian SV. Prevalence of girl and boy child marriage across states and Union Territories in India, 1993–2021: a repeated cross-sectional study. *Lancet Glob Health* 2023; published online Dec 15. https://doi.org/10.1016/S2214-109X(23)00470-9.

Gausman J., Kim R., Kumar A., Ravi S., Subramanian S.V. "Prevalence of Girl and Boy Child Marriage: Subnational Variation across States and Union Territories in India, 1993-2021"

Supplementary Material

Table of Contents

Appendix S1: Methodology used to extract data from unmarried women from household member listing in NFHS-1 and NFHS-2 2

Appendix S2: Methodology used for calculating the population headcount of men and women married as children 3

Appendix S3: Completed STROBE Checklist 4

Figure S1: Relationship between Standardized Absolute Change (1993-2021 for women and 2006-2021 for men) and Baseline (1993 for women and 2006 for men) for Girl and Boy Child Marriage across States/Union Territories of India 9

Figure S2: Status of States on Girl and Boy Child Marriage Prevalence from 1993 to 2021 10

Figure S3: Rank Order Change in Prevalence by State for Girl Child Marriage (1993-2021) and Boy Child Marriage (2006-2016) 11

Figure S4a: Relationship between the prevalence and headcount burden of girl child marriage, 2021 12

Figure S4b: Relationship between the prevalence and headcount burden of boy child marriage, 2021 13

Table S1: Study Sample Size Selection from the five National Family Health Surveys, 1993-2021 14

Table S2: Sample (*n*) and Percentage Distribution (%) Across Demographic and Socioeconomic Characteristics, India, 1993, 1999, 2006, 2016 and 2021 15

Table S3a: Girl Child Marriage Prevalence (%) and 95% Confidence Interval (CI) for India and 36 States/Union Territories, 1993-2021 17

Table S3b: Boy Child Marriage Prevalence (%) and 95% Confidence Interval (CI) for India and 36 States/Union Territories, 1993-2021 19

Table S3c: Boy Child Marriage Prevalence Per Indian Legal Definition* (%) and 95% Confidence Interval (CI) for India and 36 States/Union Territories, 1993-2021 21

Table S4a: Standardized Absolute Change (SAC) in Prevalence and 95% CI for Girl Child Marriage for States and Union Territories of India between 1993-1999, 1999-2006, 2006-2016, 2016-2021, and 1993-2021....23

Table S4b: Standardized Absolute Change (SAC) in Prevalence and 95% CI for Boy Child Marriage for States and Union Territories of India between 2006-2016, 2016-2021, and 2006-2021....24

Table S5: Estimated Headcount *N* boy child marriage for India and 36 States/Union Territories, and Percentage Share of each State/Union Territory to All-India per Indian legal Definition*, 2021 25

Table S6: National Policies and Programs, and ratified international conventions that directly or indirectly influence child marriage in India....26

Appendix S1: Methodology used to extract data from unmarried women from household member listing in NFHS-1 and NFHS-2

All ever-married women between the ages of 13-49 years (NFHS-1) and 15-49 years (NFHS-2) were eligible to participate in the women's survey module, while in NFHS-3, -4, and -5, all women aged between 15-49 years were eligible to participate. As a result, estimates of age at first marriage are not comparable across survey waves due to differences in the denominator. To ensure definitional consistency, data on unmarried women aged 20 to 24 years were extracted from the household schedule for NFHS-1 and -2. The household schedule was used to list all usual residents and visitors of each sample household – with the primary function of identifying all women eligible for the women's survey module. Basic information was collected on the age, sex, marital status, education, occupation, and relationship to the head of the household. From the household schedules, we identified all never-married women aged 20-24 years and appended the resulting observations to the women's dataset to be included in the analysis.

Appendix S2: Methodology used for calculating the population headcount of men and women married as children

To calculate the headcount, we followed IPUMS POPWT methodology.[1] To derive an estimated population headcount from women in 1993, men in 2006, and men and women in 2021, we used the 1992 India Census Data and the Census 2011 Population Projections published in July 2020.[2,3] From these tabulations, there were 36,958,481women aged 20-24 years in India in 1993 and 60,474,000 women aged 20-24 years in 2021. There were 54,642,000 men aged 20-24 years in 2006 and 66,770,000 men aged 20-24 years in 2021.

Using the population estimates for both men and women, we used the formula given by IPUMS to derive the population weight (POPWT) which was used to calculate the headcount.

$$popwt = \left(\frac{wt}{sum(wt)}\right) \times Pop$$

- "*wt*" represents the individual weight divided by 1,000,000 in the DHS microdata.
- "*sum(wt)*" represents the total value of wt
- "*Pop*" represents the total all-India estimated population of women aged 20-24 years in 1993 or 2021, or men aged 20-24 years in 2006 2021.

By weighting the data using popwt instead of the given individual weights, we derived the headcount of women and men subjected to child marriage per Indian law in all-India and its states/union territories.

References

1. IPUMS DHS. POPWT Expansion factor. [Internet]. September 9, 2022. Available from: https://www.idhsdata.org/idhs/population_weights.shtml
2. National Commission on Population, Ministry of Health and Family Welfare, Government of India. Population Projections for India and States 2011-2036: Report of the Technical Group on Population Projections. [Internet]. 2020: Available from:https://main.mohfw.gov.in/sites/default/files/Population%20Projection%20Report%202011-2036%20-%20upload_compressed_0.pdf
3. National Commission on Population, Office of the Registrar General and Census Commissioner, Government of India. Population Projections for India and States 2001-2026: Report of the Technical Group on Population Projections Constituated by the National Commission on Population. [Internet]. 2006: Available from: http://statehealthsocietybihar.org/survey_reports/Population_Projection_Report_2006.pdf

Appendix S3: Completed STROBE Checklist

STROBE Statement—checklist of items that should be included in reports of observational studies

	Item No.	Recommendation	Page No.
**Title and abstract**	1	(*a*) Indicate the study's design with a commonly used term in the title or the abstract	2
		(*b*) Provide in the abstract an informative and balanced summary of what was done and what was found	2
**Introduction**			
Background/rationale	2	Explain the scientific background and rationale for the investigation being reported	5
Objectives	3	State specific objectives, including any prespecified hypotheses	5
**Methods**			
Study design	4	Present key elements of study design early in the paper	5-6
Setting	5	Describe the setting, locations, and relevant dates, including periods of recruitment, exposure, follow-up, and data collection	5
Participants	6	(*a*) *Cohort study*—Give the eligibility criteria, and the sources and methods of selection of participants. Describe methods of follow-up<br><br>*Case-control study*—Give the eligibility criteria, and the sources and methods of case ascertainment and control selection. Give the rationale for the choice of cases and controls<br><br>*Cross-sectional study*—Give the eligibility criteria, and the sources and methods of selection of participants	6
		(*b*) *Cohort study*—For matched studies, give matching criteria and number of exposed and unexposed	

		Case-control study—For matched studies, give matching criteria and the number of controls per case	
Variables	7	Clearly define all outcomes, exposures, predictors, potential confounders, and effect modifiers. Give diagnostic criteria, if applicable	6
Data sources/ measurement	8*	For each variable of interest, give sources of data and details of methods of assessment (measurement). Describe comparability of assessment methods if there is more than one group	*6*
Bias	9	Describe any efforts to address potential sources of bias	10
Study size	10	Explain how the study size was arrived at	6

Continued on next page

Quantitative variables	11	Explain how quantitative variables were handled in the analyses. If applicable, describe which groupings were chosen and why	6
Statistical methods	12	(*a*) Describe all statistical methods, including those used to control for confounding	6
		(*b*) Describe any methods used to examine subgroups and interactions	NA
		(*c*) Explain how missing data were addressed	6
		(*d*) *Cohort study*—If applicable, explain how loss to follow-up was addressed<br>*Case-control study*—If applicable, explain how matching of cases and controls was addressed<br>*Cross-sectional study*—If applicable, describe analytical methods taking account of sampling strategy	6
		(*e*) Describe any sensitivity analyses	NA
**Results**			
Participants	13*	(a) Report numbers of individuals at each stage of study—eg numbers potentially eligible, examined for eligibility, confirmed eligible, included in the study, completing follow-up, and analysed	6,7
		(b) Give reasons for non-participation at each stage	NA
		(c) Consider use of a flow diagram	6
Descriptive data	14*	(a) Give characteristics of study participants (eg demographic, clinical, social) and information on exposures and potential confounders	7
		(b) Indicate number of participants with missing data for each variable of interest	6
		(c) *Cohort study*—Summarise follow-up time (eg, average and total amount)	
Outcome data	15*	*Cohort study*—Report numbers of outcome events or summary measures over time	

		Case-control study—Report numbers in each exposure category, or summary measures of exposure	
		Cross-sectional study—Report numbers of outcome events or summary measures	*7*
Main results	16	(*a*) Give unadjusted estimates and, if applicable, confounder-adjusted estimates and their precision (eg, 95% confidence interval). Make clear which confounders were adjusted for and why they were included	7-9
		(*b*) Report category boundaries when continuous variables were categorized	NA
		(*c*) If relevant, consider translating estimates of relative risk into absolute risk for a meaningful time period	NA

Continued on next page

Other analyses	17	Report other analyses done—eg analyses of subgroups and interactions, and sensitivity analyses	NA
**Discussion**			
Key results	18	Summarise key results with reference to study objectives	9
Limitations	19	Discuss limitations of the study, taking into account sources of potential bias or imprecision. Discuss both direction and magnitude of any potential bias	11
Interpretation	20	Give a cautious overall interpretation of results considering objectives, limitations, multiplicity of analyses, results from similar studies, and other relevant evidence	11
Generalisability	21	Discuss the generalisability (external validity) of the study results	11
**Other information**			
Funding	22	Give the source of funding and the role of the funders for the present study and, if applicable, for the original study on which the present article is based	2

*Give information separately for cases and controls in case-control studies and, if applicable, for exposed and unexposed groups in cohort and cross-sectional studies.

Note: An Explanation and Elaboration article discusses each checklist item and gives methodological background and published examples of transparent reporting. The STROBE checklist is best used in conjunction with this article (freely available on the Web sites of PLoS Medicine at http://www.plosmedicine.org/, Annals of Internal Medicine at http://www.annals.org/, and Epidemiology at http://www.epidem.com/). Information on the STROBE Initiative is available at www.strobe-statement.org.

Figure S1: Relationship between Standardized Absolute Change (1993-2021 for women and 2006-2021 for men) and Baseline (1993 for women and 2006 for men) for Girl and Boy Child Marriage across States/Union Territories of India

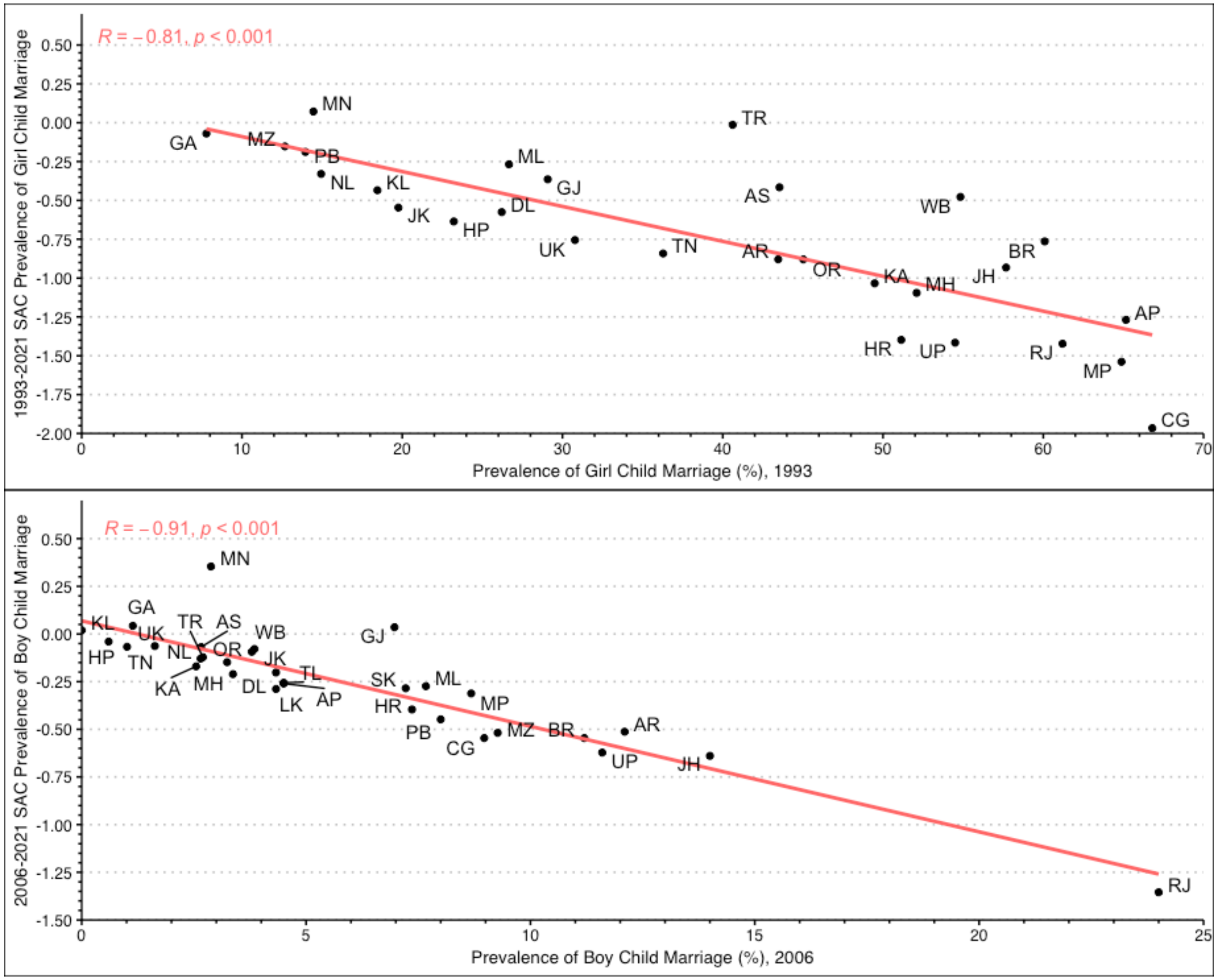

Figure S2: Status of States on Girl and Boy Child Marriage Prevalence from 1993 to 2021

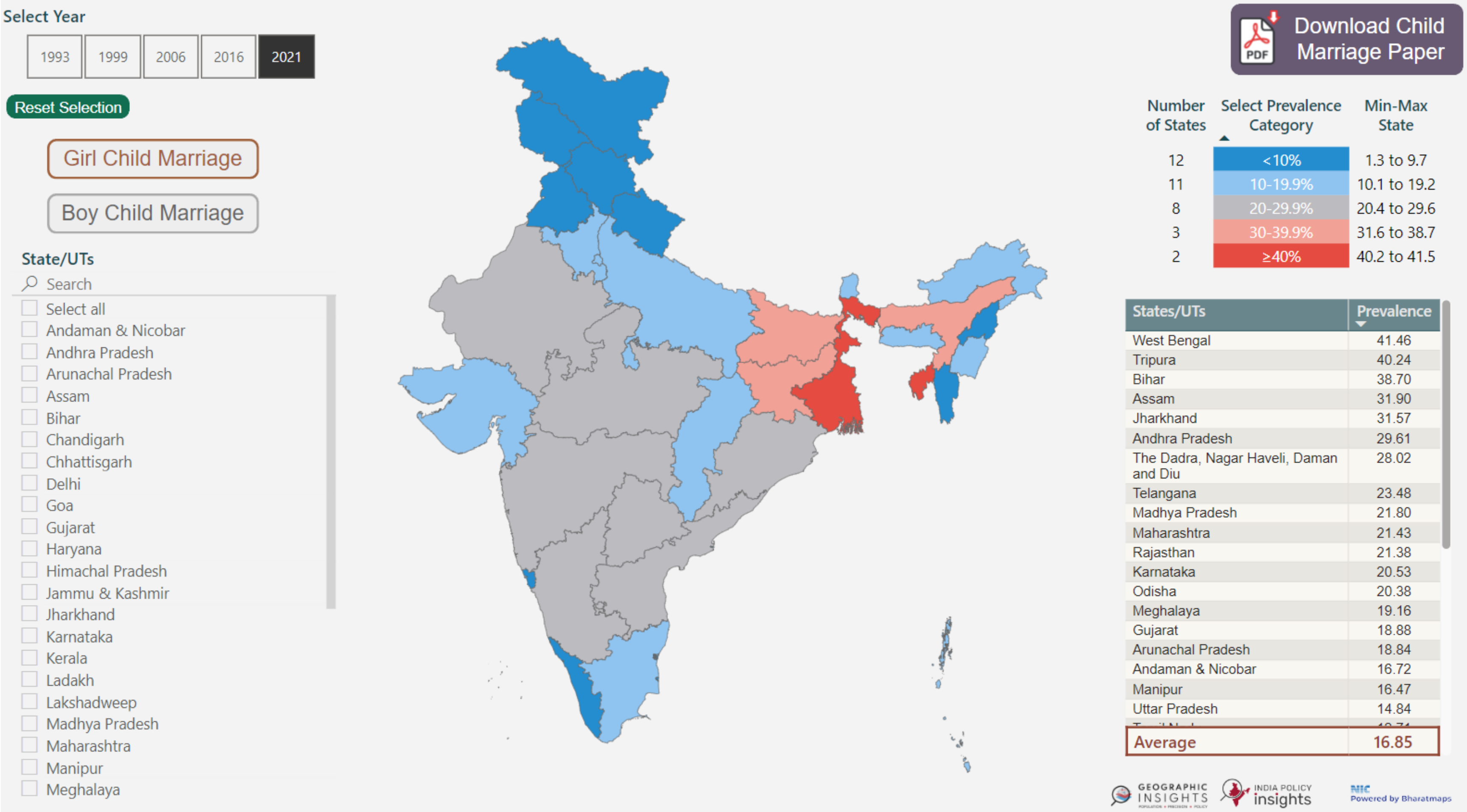

Note: See https://geographicinsights.iq.harvard.edu/State-Child-Marriage

Figure S3: Rank Order Change in Prevalence by State for Girl Child Marriage (1993-2021) and Boy Child Marriage (2006-2016)

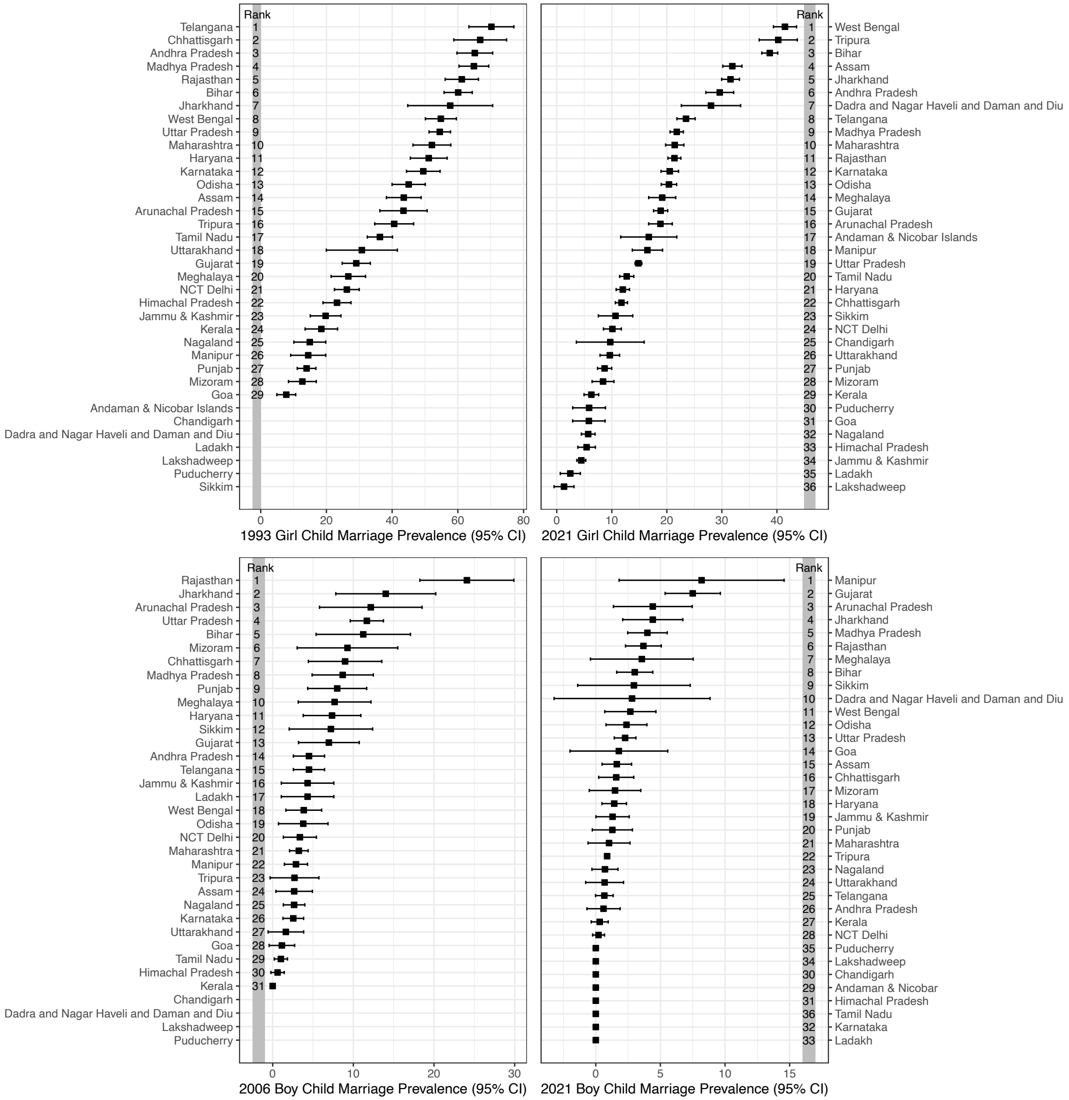

Figure S4a: Relationship between the prevalence and headcount burden of girl child marriage, 2021

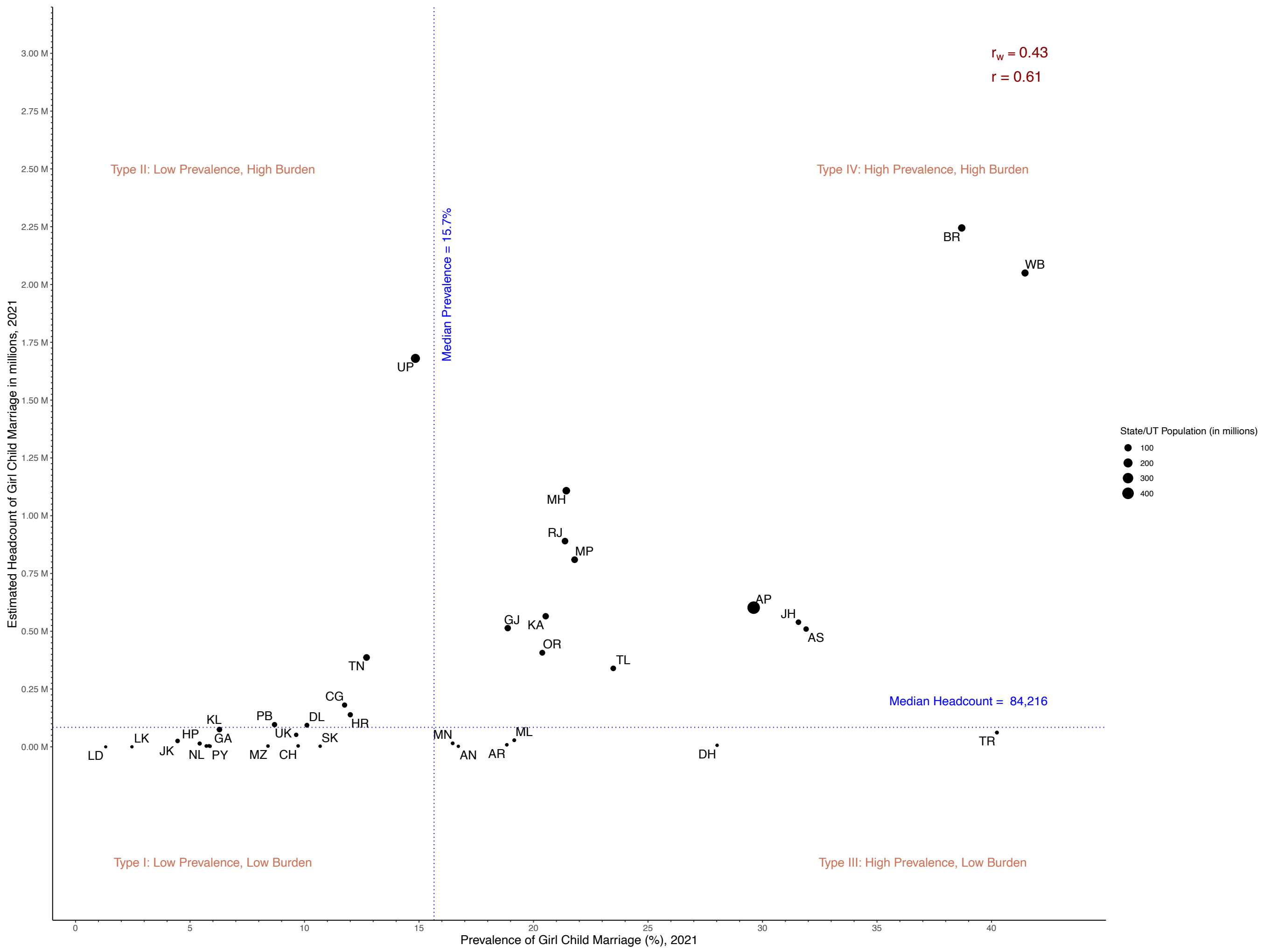

Figure S4b: Relationship between the prevalence and headcount burden of boy child marriage, 2021

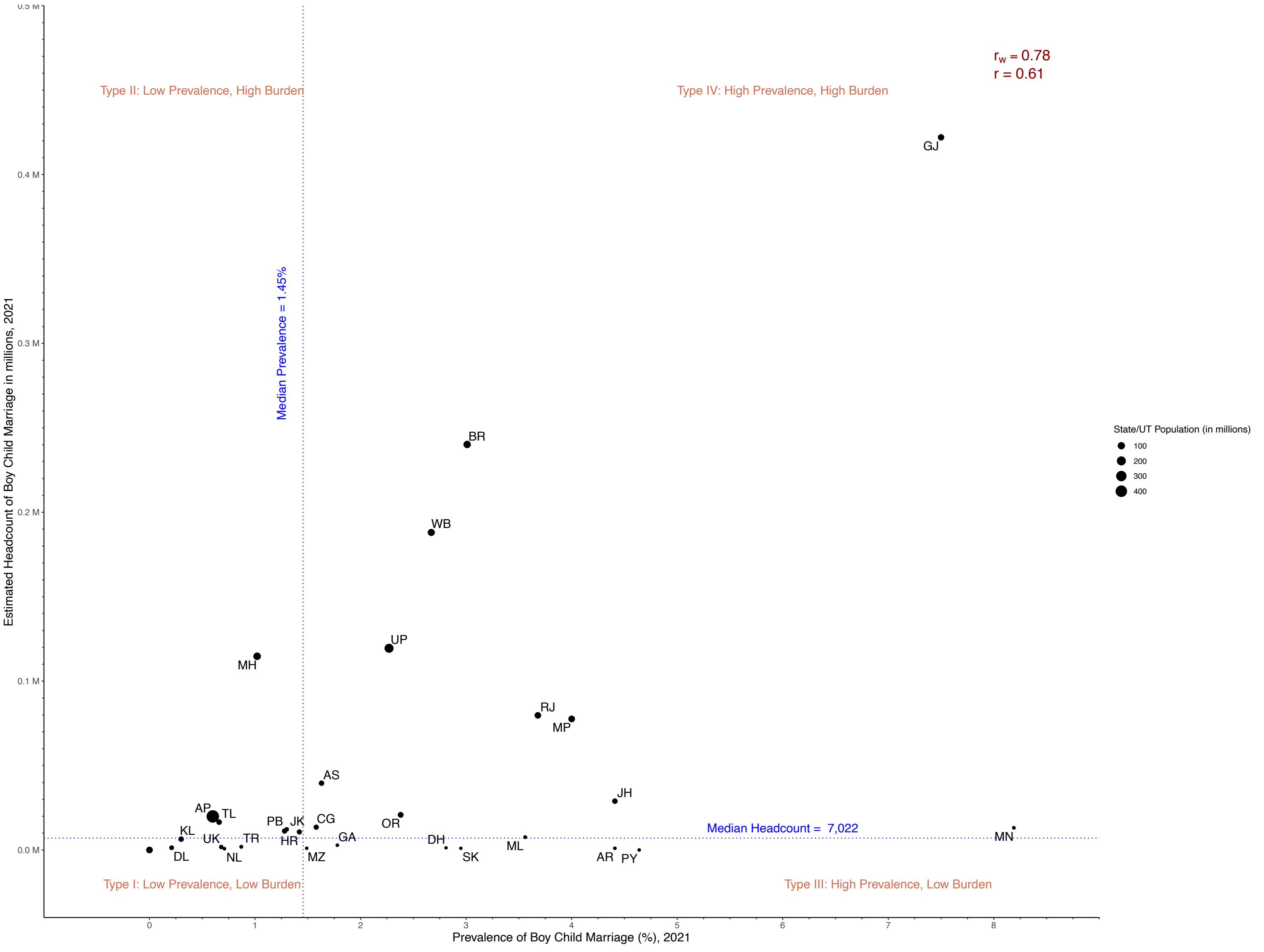

Table S1: Study Sample Size Selection from the five National Family Health Surveys, 1993-2021

Survey round Year	Data source	Sample Size based on Inclusion Criteria n	Missing age at first marriage n	Missing Geographical Data	Final Study Sample Size n
**Women**					
NFHS-1 1992-93	Women's Survey	6,267	4	38	6,225
NFHS-1 1992-93	Household Roster	17,218	0	13	17,205
NFHS-2 1998-99	Women's Survey	6,856	0	0	6,856
NFHS-2 1998-99	Household Roster	15,973	0	0	15,973
NFHS-3 2005-06	Women's survey	22,807	0	0	22,807
NFHS-4 2015-16	Women's survey	122,955	0	0	122,955
NFHS-5 2019-21	Women's survey	118,700	0	0	118,700
**All waves**		**310,776**	**4**	**51**	**310,721**
**Men**					
NFHS-3 2005-06	Men's Survey	12,460	0	0	12,440
NFHS-4 2015-16	Men's Survey	16,630	64	0	16,563
NFHS-5 2019-21	Men's Survey	14,413	0	0	14,413
**All waves**		**43,503**	**64**	**0**	**43,436**

Table S2: Sample (*n*) and Percentage Distribution (%) Across Demographic and Socioeconomic Characteristics, India, 1993, 1999, 2006, 2016 and 2021

	**1993**		**1999**		**2006**		**2016**		**2021**	
	n	%	*n*	%	*n*	%	*n*	%	*n*	%
**Women (Total)**	23,430	100	22,829	100	22,807	100	122,955	100	118,700	100
Age										
20 years	6,220	21·21	5,918	24·16	5,380	23·59	27,789	22·38	26,061	21·72
21 years	4,132	17·86	3,927	18·37	4,291	18·81	21,856	17·88	21,876	18·67
22 years	4,858	21·07	4,819	20·99	4,809	21·09	26,837	21·93	24,912	21·17
23 years	3,969	19·73	4,115	18·92	4,331	18·99	23,601	19·12	22,976	19·17
24 years	4,251	20·14	4,050	17·56	3,996	17·52	22,872	18·69	22,875	19·28
Place of residence										
Urban	7,679	28·14	7,601	28·85	10,429	32·78	35,236	33·74	27,632	30·61
Rural	15,751	71·86	15,228	71·15	12,378	67·22	87,719	66·26	91,068	69·39
Social Caste										
Other	··	79·01	8,647	18·06	7,622	18·9	23,834	20·97	20,759	22·83
OBC	17,837	··	6,256	9·03	7,136	8·33	48,685	9·5	45,868	9·71
Scheduled Caste	2,605	11·72	3,726	31·87	3,823	39·07	22,718	43·19	24,018	42·93
Scheduled Tribe	2,988	9·21	3,081	35·61	3,156	30·54	21,910	21·73	21,829	19·27
Not reported/ Don't Know	··	··	1,119	5·44	185	3·16	5,808	4·62	6,226	5·62
Educational Attainment										
No formal schooling	10,034	49·87	7,789	38·82	5,264	38·82	17,817	13·66	10,612	8·73
Primary (one to five years)	4,591	19·19	3,321	15	2,862	15	13,129	10·57	8,647	7·3
Secondary (six to 12 years)	6,650	23·51	7,088	28·6	11,058	28·6	63,808	50·21	65,261	52·93
College education (more than 12 years)	2,057	7·06	4,626	17·58	3,622	17·58	28,201	25·56	34,180	31·04
Missing	98	0·37	5	0	1	0	0	0	0	0
**Men (Total)**	··	··	··	··	12,460	100	16,563	100	14,413	100
Age										
20 years	··	··	··	··	2,879	23·11	3,729	22·51	3,215	22·31
21 years	··	··	··	··	2,446	19·63	3,096	18·69	2,679	18·59
22 years	··	··	··	··	2,743	22·01	3,548	21·42	2,951	20·47
23 years	··	··	··	··	2,220	17·82	3,066	18·51	2,741	19·02
24 years	··	··	··	··	2,172	17·43	3,124	18·86	2,827	19·61
Place of residence										
Urban	··	··	··	··	6,876	55·18	5,276	31·85	3,749	26·01
Rural	··	··	··	··	5,584	44·82	11,287	68·15	10,664	73·99

Social Caste										
Other	··	··	··	··	3,906	32·17	3,123	19·84	2,525	18·38
Other/ OBC	··	··	··	··	4,490	36·98	6,534	41·52	5,678	41·34
Scheduled Caste	··	··	··	··	2,221	18·29	3,173	20·16	2,872	20·91
Scheduled Tribe	··	··	··	··	1,458	12·01	2,856	18·15	2,614	19·03
Not reported/ Don't Know	··	··	··	··	67	0·55	51	0·32	47	0·34
Educational Attainment										
No formal schooling	··	··	··	··	1,159	9·3	1,058	6·39	741	5·14
Primary (one to five years)	··	··	··	··	1,584	12·71	1,526	9·21	904	6·27
Secondary (six to 12 years)	··	··	··	··	7,174	57·58	9,375	56·6	8,146	56·52
College education (more than 12 years)	··	··	··	··	2,539	20·38	4,604	27·8	4,622	32·07
Missing	··	··	··	··	4	0·03	0	0	0	0

Note: Proportions are weighted for survey design

Table S3a: Girl Child Marriage Prevalence (%) and 95% Confidence Interval (CI) for India and 36 States/Union Territories, 1993-2021

	1993		1999		2006		2016		2021	
	**%**	**95% CI**	**%**	**95% CI**	**%**	**95% CI**	**%**	**95% CI**	**%**	**95% CI**
India	49·42	[48·08 - 50·77]	45·10	[44·29 - 45·91]	44·50	[43·28 - 45·74]	25·28	[24·86 - 25·70]	22·26	[21·87 - 22·66]
States										
Andhra Pradesh	65·16	[59·72 - 70·59]	65·64	[61·55 - 69·73]	54·71	[50·22 - 59·20]	32·65	[30·00 - 35·31]	29·61	[27·09 - 32·14]
Arunachal Pradesh	43·46	[36·26 - 50·65]	26·67	[22·11 - 31·22]	40·64	[33·76 - 47·53]	27·27	[24·91 - 29·63]	18·84	[16·69 - 20·98]
Assam	43·54	[38·26 - 48·82]	36·33	[32·90 - 39·76]	37·95	[32·66 - 43·25]	32·39	[30·71 - 34·07]	31·90	[30·16 - 33·65]
Bihar	60·09	[55·80 - 64·37]	58·42	[55·60 - 61·23]	60·28	[55·15 - 65·42]	39·04	[37·71 - 40·37]	38·70	[37·25 - 40·15]
Chhattisgarh	66·80	[58·78 - 74·83]	55·82	[49·34 - 62·30]	51·83	[47·21 - 56·45]	20·05	[18·50 - 21·60]	11·75	[10·65 - 12·85]
Goa	7·78	[4·91 - 10·65]	8·11	[5·25 - 10·98]	11·72	[8·44 - 15·01]	9·78	[4·49 - 15·07]	5·83	[2·88 - 8·78]
Gujarat	29·08	[24·79 - 33·36]	35·66	[32·53 - 38·79]	33·46	[28·09 - 38·84]	22·62	[20·83 - 24·40]	18·88	[17·59 - 20·16]
Haryana	51·14	[45·54 - 56·74]	36·85	[33·05 - 40·65]	39·81	[34·87 - 44·76]	18·45	[17·04 - 19·87]	12·00	[10·79 - 13·21]
Himachal Pradesh	23·22	[18·96 - 27·48]	9·23	[7·12 - 11·35]	12·34	[9·37 - 15·30]	8·59	[6·78 - 10·41]	5·42	[3·83 - 7·02]
Jharkhand	57·68	[44·76 - 70·59]	56·30	[51·41 - 61·19]	61·16	[55·35 - 66·96]	37·03	[35·45 - 38·61]	31·57	[29·95 - 33·19]
Karnataka	49·48	[44·38 - 54·59]	41·77	[38·75 - 44·79]	41·22	[36·90 - 45·54]	23·16	[21·37 - 24·96]	20·53	[18·94 - 22·12]
Kerala	18·45	[13·51 - 23·40]	15·65	[12·79 - 18·52]	15·43	[11·67 - 19·18]	7·67	[5·81 - 9·54]	6·29	[4·96 - 7·62]
Madhya Pradesh	64·88	[60·36 - 69·41]	61·89	[59·11 - 64·67]	53·01	[48·62 - 57·40]	30·11	[29·07 - 31·15]	21·80	[20·59 - 23·00]
Maharashtra	52·10	[46·34 - 57·86]	44·08	[40·94 - 47·23]	38·97	[35·29 - 42·66]	25·04	[23·10 - 26·99]	21·43	[19·77 - 23·09]
Manipur	14·46	[9·11 - 19·80]	9·93	[7·26 - 12·61]	12·73	[9·80 - 15·66]	13·08	[11·47 - 14·69]	16·47	[13·72 - 19·22]
Meghalaya	26·66	[21·42 - 31·90]	24·83	[19·96 - 29·69]	24·49	[18·64 - 30·35]	16·45	[14·05 - 18·85]	19·16	[16·71 - 21·61]
Mizoram	12·68	[8·45 - 16·91]	10·81	[7·60 - 14·01]	20·58	[15·26 - 25·90]	10·27	[8·22 - 12·33]	8·41	[6·42 - 10·39]
Nagaland	14·94	[10·09 - 19·80]	20·77	[15·85 - 25·68]	21·07	[17·30 - 24·83]	13·18	[11·21 - 15·14]	5·71	[4·47 - 6·96]
Odisha	45·03	[39·98 - 50·07]	38·14	[35·17 - 41·10]	36·34	[30·97 - 41·72]	21·15	[19·84 - 22·45]	20·38	[18·98 - 21·78]
Punjab	13·96	[11·13 - 16·79]	11·86	[9·54 - 14·17]	19·39	[16·23 - 22·54]	7·28	[6·36 - 8·21]	8·69	[7·40 - 9·98]
Rajasthan	61·20	[56·13 - 66·27]	59·90	[57·38 - 62·42]	57·11	[53·15 - 61·07]	29·83	[28·64 - 31·01]	21·38	[20·20 - 22·55]
Sikkim			20·52	[16·31 - 24·73]	30·12	[24·90 - 35·34]	14·49	[11·72 - 17·27]	10·68	[7·56 - 13·81]
Tamil Nadu	36·28	[32·45 - 40·10]	23·32	[20·75 - 25·89]	21·54	[18·20 - 24·87]	15·80	[14·29 - 17·30]	12·71	[11·42 - 14·00]
Telangana	70·21	[63·37 - 77·05]	58·82	[53·51 - 64·12]	54·71	[50·22 - 59·20]	25·35	[22·42 - 28·28]	23·48	[21·83 - 25·14]
Tripura	40·62	[34·70 - 46·54]	35·67	[30·16 - 41·17]	40·96	[34·69 - 47·23]	32·31	[28·71 - 35·92]	40·24	[36·78 - 43·70]
Uttar Pradesh	54·50	[51·23 - 57·77]	57·43	[55·21 - 59·65]	53·10	[50·16 - 56·04]	18·63	[17·94 - 19·33]	14·84	[14·22 - 15·47]
Uttarakhand	30·78	[19·98 - 41·59]	23·29	[17·71 - 28·88]	22·56	[17·58 - 27·55]	13·95	[12·51 - 15·38]	9·64	[7·86 - 11·42]
West Bengal	54·83	[50·12 - 59·54]	45·33	[41·99 - 48·67]	53·34	[49·66 - 57·02]	40·86	[38·72 - 43·00]	41·46	[39·36 - 43·56]
Union Territories (UTs)										
NCT Delhi	26·21	[22·45 - 29·97]	17·94	[14·89 - 20·99]	21·20	[16·55 - 25·85]	14·04	[9·91 - 18·17]	10·11	[8·48 - 11·73]
Jammu & Kashmir	19·76	[15·10 - 24·42]	20·11	[17·21 - 23·00]	14·03	[10·72 - 17·35]	8·46	[7·44 - 9·48]	4·46	[3·65 - 5·27]
Ladakh					14·03	[10·72 - 17·35]	4·85	[2·34 - 7·35]	2·46	[0·64 - 4·29]

Andaman & Nicobar	17·10	[11·75 - 22·45]	16·72	[11·62 - 21·81]
Chandigarh	11·92	[4·65 - 19·18]	9·72	[3·56 - 15·88]
Dadra and Nagar Haveli and Daman and Diu	27·30	[21·51 - 33·10]	28·02	[22·63 - 33·40]
Lakshadweep	1·88	[0·74 - 3·03]	1·32	[-0·49 - 3·13]
Puducherry	10·66	[5·94 - 15·38]	5·87	[2·90 - 8·85]

Table S3b: Boy Child Marriage Prevalence (%) and 95% Confidence Interval (CI) for India and 36 States/Union Territories, 1993-2021

	2006		2016		2021	
	**%**	**95% CI**	**%**	**95% CI**	**%**	**95% CI**
India	7·11	[6·37 - 7·86]	3·38	[2·99 - 3·78]	2·22	[1·77 - 2·67]
			States			
Andhra Pradesh	4·50	[2·57 - 6·43]	2·95	[0·06 - 5·84]	0·60	[-0·69 - 1·88]
Arunachal Pradesh	12·17	[5·81 - 18·52]	5·48	[2·26 - 8·70]	4·41	[1·36 - 7·46]
Assam	2·67	[0·41 - 4·93]	2·93	[1·31 - 4·54]	1·63	[0·48 - 2·77]
Bihar	11·23	[5·38 - 17·08]	4·63	[2·88 - 6·39]	3·01	[1·62 - 4·41]
Chhattisgarh	8·98	[4·42 - 13·53]	1·24	[0·41 - 2·07]	1·58	[0·22 - 2·94]
Goa	1·14	[-0·44 - 2·72]	0·00	[0·00 - 0·00]	1·78	[-2·00 - 5·57]
Gujarat	6·97	[3·21 - 10·73]	9·07	[6·72 - 11·42]	7·50	[5·37 - 9·64]
Haryana	7·36	[3·79 - 10·93]	3·75	[1·85 - 5·64]	1·42	[0·47 - 2·38]
Himachal Pradesh	0·61	[-0·22 - 1·44]	0·39	[-0·19 - 0·97]	0·00	[0·00 - 0·00]
Jharkhand	14·02	[7·83 - 20·22]	5·09	[3·08 - 7·10]	4·41	[2·08 - 6·74]
Karnataka	2·56	[1·28 - 3·84]	1·60	[-0·45 - 3·66]	0·00	[0·00 - 0·00]
Kerala	0·00	[0·00 - 0·00]	0·00	[0·00 - 0·00]	0·30	[-0·35 - 0·96]
Madhya Pradesh	8·68	[4·89 - 12·48]	4·72	[3·44 - 6·00]	4·00	[2·47 - 5·52]
Maharashtra	3·25	[2·10 - 4·40]	1·12	[0·21 - 2·03]	1·02	[-0·60 - 2·65]
Manipur	2·89	[1·46 - 4·32]	1·38	[-0·03 - 2·78]	8·19	[1·80 - 14·57]
Meghalaya	7·68	[3·17 - 12·19]	4·83	[0·24 - 9·42]	3·56	[-0·41 - 7·54]
Mizoram	9·27	[3·03 - 15·51]	1·87	[0·31 - 3·43]	1·49	[-0·51 - 3·48]
Nagaland	2·66	[1·33 - 3·99]	1·87	[-0·29 - 4·03]	0·71	[-0·30 - 1·72]
Odisha	3·79	[0·72 - 6·86]	2·23	[0·96 - 3·50]	2·38	[0·79 - 3·96]
Punjab	8·00	[4·34 - 11·66]	0·92	[-0·10 - 1·94]	1·28	[-0·27 - 2·83]
Rajasthan	24·08	[18·25 - 29·91]	7·18	[5·18 - 9·17]	3·68	[2·30 - 5·07]
Sikkim	7·22	[2·05 - 12·39]	2·77	[-0·95 - 6·49]	2·95	[-1·40 - 7·31]
Tamil Nadu	1·02	[0·20 - 1·84]	0·75	[-0·02 - 1·53]	0·00	[0·00 - 0·00]
Telangana	2·71	[-0·32 - 5·73]	1·40	[-0·63 - 3·44]	0·66	[-0·02 - 1·34]
Tripura	11·68	[2·57 - 6·43]	2·88	[-0·56 - 6·32]	0·87	[0·75 - 1·00]
Uttar Pradesh	1·64	[9·62 - 13·73]	3·83	[2·95 - 4·71]	2·27	[1·43 - 3·10]
Uttarakhand	3·85	[-0·57 - 3·84]	0·78	[-0·27 - 1·82]	0·68	[-0·78 - 2·15]
West Bengal	4·50	[1·64 - 6·07]	3·47	[1·37 - 5·57]	2·67	[0·70 - 4·65]
			Union Territories (UTs)			
NCT Delhi	3·37	[1·33 - 5·42]	3·58	[-0·91 - 8·07]	0·21	[-0·25 - 0·67]
Jammu & Kashmir	4·33	[1·07 - 7·59]	1·50	[0·59 - 2·41]	1·30	[0·01 - 2·59]

Ladakh	4·33	[1·07 - 7·59]	0·00	[0·00 - 0·00]	0·00	[0·00 - 0·00]
Andaman & Nicobar			0·43	[-0·47 - 1·32]	0·00	[0·00 - 0·00]
Chandigarh			0·00	[0·00 - 0·00]	0·00	[0·00 - 0·00]
Dadra and Nagar Haveli and Daman and Diu			1·78	[-1·11 - 4·67]	2·81	[-3·23 - 8·84]
Lakshadweep			0·00	[0·00 - 0·00]	0·00	[0·00 - 0·00]
Puducherry			6·38	[0·00 - 0·00]	4·64	[0·00 - 0·00]

Table S3c: Boy Child Marriage Prevalence Per Indian Legal Definition* (%) and 95% Confidence Interval (CI) for India and 36 States/Union Territories, 1993-2021

	2006		2016		2021	
	%	95% CI	%	95% CI	%	95% CI
India	29·27	[27·78 - 30·75]	19·16	[18·16 - 20·16]	15·49	[14·27 - 16·70]
States						
Andhra Pradesh	33·52	[29·00 - 38·03]	21·39	[13·95 - 28·83]	14·61	[8·10 - 21·11]
Arunachal Pradesh	26·93	[15·96 - 37·89]	26·85	[19·67 - 34·03]	25·13	[18·37 - 31·90]
Assam	14·68	[9·91 - 19·46]	16·97	[12·87 - 21·07]	18·12	[14·80 - 21·44]
Bihar	39·45	[30·16 - 48·74]	29·55	[25·20 - 33·90]	26·63	[22·42 - 30·83]
Chhattisgarh	39·91	[29·72 - 50·11]	21·95	[16·61 - 27·29]	14·45	[10·92 - 17·97]
Goa	6·99	[2·78 - 11·20]	6·25	[-0·26 - 12·75]	8·95	[-5·05 - 22·94]
Gujarat	29·61	[21·97 - 37·26]	27·17	[22·82 - 31·53]	24·24	[20·47 - 28·00]
Haryana	31·81	[24·98 - 38·64]	20·59	[16·31 - 24·88]	13·66	[9·94 - 17·39]
Himachal Pradesh	13·84	[6·39 - 21·28]	4·60	[2·62 - 6·58]	6·61	[2·38 - 10·83]
Jharkhand	49·73	[39·28 - 60·18]	28·91	[24·22 - 33·61]	23·26	[19·20 - 27·31]
Karnataka	12·25	[9·54 - 14·96]	7·62	[5·50 - 9·73]	4·06	[1·69 - 6·43]
Kerala	3·04	[0·13 - 5·94]	7·62	[-0·50 - 4·14]	0·84	[-0·66 - 2·35]
Madhya Pradesh	50·90	[44·78 - 57·02]	32·92	[29·93 - 35·91]	25·18	[21·84 - 28·52]
Maharashtra	14·04	[11·02 - 17·05]	10·85	[7·65 - 14·04]	8·14	[4·09 - 12·19]
Manipur	11·05	[8·18 - 13·91]	16·12	[9·74 - 22·50]	14·70	[7·13 - 22·27]
Meghalaya	26·07	[18·82 - 33·32]	16·06	[10·16 - 21·96]	19·12	[12·86 - 25·37]
Mizoram	30·92	[21·41 - 40·43]	12·65	[7·95 - 17·35]	7·23	[2·74 - 11·72]
Nagaland	17·12	[13·09 - 21·15]	7·27	[3·13 - 11·40]	5·18	[1·61 - 8·75]
Odisha	18·11	[12·52 - 23·69]	12·71	[9·24 - 16·17]	10·30	[7·13 - 13·46]
Punjab	29·08	[21·67 - 36·50]	11·16	[6·75 - 15·57]	8·41	[4·98 - 11·84]
Rajasthan	49·07	[41·48 - 56·66]	30·46	[26·88 - 34·04]	26·43	[22·94 - 29·92]
Sikkim	23·90	[15·22 - 32·58]	10·58	[4·94 - 16·22]	5·44	[1·52 - 9·37]
Tamil Nadu	8·80	[6·02 - 11·59]	8·47	[5·32 - 11·63]	4·82	[2·30 - 7·33]
Telangana	33·52	[29·00 - 38·03]	15·50	[9·00 - 22·00]	13·56	[7·56 - 19·56]
Tripura	12·36	[6·25 - 18·47]	12·43	[4·74 - 20·12]	19·23	[8·77 - 29·69]
Uttar Pradesh	45·76	[42·04 - 49·48]	24·80	[22·31 - 27·28]	19·80	[17·01 - 22·59]
Uttarakhand	18·32	[11·23 - 25·41]	14·16	[9·30 - 19·01]	11·11	[5·60 - 16·63]
West Bengal	24·60	[18·93 - 30·27]	19·60	[14·19 - 25·01]	20·18	[15·11 - 25·26]
Union Territories (UTs)						
NCT Delhi	17·11	[11·01 - 23·20]	21·85	[11·57 - 32·13]	8·45	[4·45 - 12·45]
Jammu & Kashmir	16·04	[9·79 - 22·30]	10·02	[6·73 - 13·32]	7·61	[4·29 - 10·94]
Ladakh	16·04	[9·79 - 22·30]	8·36	[-0·71 - 17·44]	16·33	[2·04 - 30·62]

Andaman & Nicobar	8·52	[1·14 - 15·90]	7·08	[-3·43 - 17·59]
Chandigarh	0·00	[0·00 - 0·00]	0·00	[0·00 - 0·00]
Dadra and Nagar Haveli and Daman and Diu	28·37	[18·39 - 38·36]	7·14	[0·27 - 14·00]
Lakshadweep	0·00	[0·00 - 0·00]	0·00	[0·00 - 0·00]
Puducherry	6·38	[-4·89 - 17·65]	4·64	[-4·40 - 13·69]

***Note:** Boy child marriage per the Indian legal definition is defined as boys married prior to their 21st birthday. We calculate estimates among men aged 23-27 years of age for consistency with the international definition.

Table S4a: Standardized Absolute Change (SAC) in Prevalence and 95% CI for Girl Child Marriage for States and Union Territories of India between 1993-1999, 1999-2006, 2006-2016, 2016-2021, and 1993-2021

	**1993-2021**			**1993-1999**			**1999-2006**			**2006-2016**			**2016-2021**		
	SAC	95% CI	p-value	SAC	95% CI	p-value	SAC	95% CI	p-value	SAC	95% CI	p-value	SAC	95% CI	p-value
All India	-0.97	[-1.02 - -0.92]	<0.001	-0.72	[-0.98 - -0.46]	<0.001	-0.08	[-0.29 - 0.13]	0.43	-1.92	[-2.05 - -1.79]	<0.001	-0.60	[-0.72 - -0.48]	<0.001
Andhra Pradesh	-1.27	[-1.46 - -1.08]	<0.001	0.08	[-0.93 - 1.09]	0.88	-1.56	[-2.43 - -0.69]	0.00	-2.21	[-2.74 - -1.68]	<0.001	-0.61	[-1.35 - 0.14]	0.11
Arunachal Pradesh	-0.88	[-1.14 - -0.62]	<0.001	-2.80	[-4.18 - -1.41]	<0.001	2.00	[0.81 - 3.19]	0.00	-1.34	[-2.07 - -0.60]	<0.001	-1.69	[-2.33 - -1.04]	0.00
Assam	-0.42	[-0.61 - -0.22]	<0.001	-1.20	[-2.25 - -0.15]	0.02	0.23	[-0.67 - 1.14]	0.62	-0.56	[-1.12 - 0.01]	0.05	-0.10	[-0.59 - 0.40]	0.70
Bihar	-0.76	[-0.92 - -0.61]	<0.001	-0.28	[-1.08 - 0.52]	0.50	0.27	[-0.58 - 1.11]	0.54	-2.12	[-2.66 - -1.58]	<0.001	-0.07	[-0.48 - 0.34]	0.74
Chhattisgarh	-1.97	[-2.17 - -1.76]	<0.001	-1.83	[-3.21 - -0.45]	0.01	-0.57	[-1.69 - 0.55]	0.32	-3.18	[-3.69 - -2.67]	<0.001	-1.66	[-2.06 - -1.26]	<0.001
Goa	-0.07	[-0.22 - 0.08]	0.36	0.06	[-0.60 - 0.71]	0.87	0.52	[-0.12 - 1.15]	0.11	-0.19	[-0.85 - 0.46]	0.56	-0.79	[-2.07 - 0.49]	0.23
Gujarat	-0.36	[-0.52 - -0.21]	<0.001	1.10	[0.22 - 1.97]	0.01	-0.31	[-1.20 - 0.57]	0.49	-1.08	[-1.65 - -0.51]	<0.001	-0.75	[-1.20 - -0.30]	<0.001
Haryana	-1.40	[-1.60 - -1.20]	<0.001	-2.38	[-3.50 - -1.26]	<0.001	0.42	[-0.45 - 1.30]	0.34	-2.14	[-2.65 - -1.62]	<0.001	-1.29	[-1.68 - -0.90]	<0.001
Himachal Pradesh	-0.64	[-0.80 - -0.47]	<0.001	-2.33	[-3.12 - -1.54]	<0.001	0.44	[-0.08 - 0.97]	0.10	-0.37	[-0.73 - -0.02]	0.04	-0.63	[-1.13 - -0.13]	0.01
Jammu & Kashmir	-0.55	[-0.71 - -0.38]	<0.001	0.06	[-0.85 - 0.97]	0.90	-0.87	[-1.50 - -0.24]	0.01	-0.56	[-0.91 - -0.20]	<0.001	-0.80	[-1.08 - -0.52]	<0.001
Jharkhand	-0.93	[-1.45 - -0.41]	<0.001	-0.23	[-2.77 - 2.31]	0.86	0.69	[-0.38 - 1.76]	0.20	-2.41	[-3.02 - -1.81]	<0.001	-1.09	[-1.57 - -0.61]	<0.001
Karnataka	-1.03	[-1.22 - -0.85]	<0.001	-1.29	[-2.26 - -0.31]	0.01	-0.08	[-0.82 - 0.66]	0.84	-1.81	[-2.28 - -1.33]	<0.001	-0.53	[-1.03 - -0.02]	0.04
Kerala	-0.43	[-0.61 - -0.25]	<0.001	-0.47	[-1.41 - 0.48]	0.33	-0.03	[-0.69 - 0.62]	0.92	-0.78	[-1.20 - -0.35]	<0.001	-0.28	[-0.76 - 0.20]	0.26
Madhya Pradesh	-1.54	[-1.70 - -1.38]	<0.001	-0.50	[-1.36 - 0.36]	0.26	-1.27	[-2.02 - -0.51]	<0.001	-2.29	[-2.76 - -1.82]	<0.001	-1.66	[-2.00 - -1.32]	<0.001
Maharashtra	-1.10	[-1.31 - -0.89]	<0.001	-1.34	[-2.42 - -0.25]	0.02	-0.73	[-1.40 - -0.06]	0.03	-1.39	[-1.82 - -0.97]	<0.001	-0.72	[-1.26 - -0.19]	0.01
Manipur	0.07	[-0.15 - 0.29]	0.52	-0.75	[-1.77 - 0.26]	0.14	0.40	[-0.18 - 0.98]	0.17	0.03	[-0.31 - 0.37]	0.84	0.68	[0.03 - 1.33]	0.04
Meghalaya	-0.27	[-0.47 - -0.06]	0.01	-0.31	[-1.49 - 0.88]	0.62	-0.05	[-1.13 - 1.04]	0.93	-0.80	[-1.44 - -0.17]	0.01	0.54	[-0.14 - 1.23]	0.12
Mizoram	-0.15	[-0.31 - 0.01]	0.06	-0.31	[-1.16 - 0.54]	0.47	1.40	[0.51 - 2.29]	<0.001	-1.03	[-1.61 - -0.45]	<0.001	-0.37	[-0.96 - 0.22]	0.21
Nagaland	-0.33	[-0.51 - -0.15]	<0.001	0.97	[-0.19 - 2.13]	0.10	0.04	[-0.85 - 0.94]	0.93	-0.79	[-1.22 - -0.36]	<0.001	-1.49	[-1.97 - -1.01]	<0.001
Nct Of Delhi	-0.58	[-0.73 - -0.42]	<0.001	-1.38	[-2.21 - -0.54]	0.00	0.47	[-0.36 - 1.29]	0.27	-0.72	[-1.37 - -0.06]	0.03	-0.79	[-1.71 - 0.14]	0.10
Odisha	-0.88	[-1.07 - -0.70]	<0.001	-1.15	[-2.10 - -0.20]	0.02	-0.26	[-1.13 - 0.61]	0.56	-1.52	[-2.08 - -0.96]	<0.001	-0.15	[-0.56 - 0.26]	0.46
Punjab	-0.19	[-0.30 - -0.08]	<0.001	-0.35	[-0.95 - 0.24]	0.25	1.08	[0.52 - 1.63]	<0.001	-1.21	[-1.54 - -0.88]	<0.001	0.28	[-0.04 - 0.60]	0.08
Rajasthan	-1.42	[-1.61 - -1.24]	<0.001	-0.22	[-1.14 - 0.70]	0.64	-0.40	[-1.07 - 0.28]	0.25	-2.73	[-3.16 - -2.30]	<0.001	-1.69	[-2.04 - -1.34]	<0.001
Sikkim	0.00	[0.00 - 0.00]	<0.001	0.00	[0.00 - 0.00]	0.00	1.37	[0.39 - 2.36]	0.01	-1.56	[-2.16 - -0.96]	<0.001	-0.76	[-1.59 - 0.07]	0.07
Tamil Nadu	-0.84	[-0.98 - -0.70]	<0.001	-2.16	[-2.92 - -1.39]	0.00	-0.25	[-0.85 - 0.34]	0.41	-0.57	[-0.94 - -0.21]	<0.001	-0.62	[-1.02 - -0.21]	<0.001
Telangana	-1.67	[-1.87 - -1.47]	<0.001	-1.90	[-3.16 - -0.64]	0.00	-0.59	[-1.58 - 0.40]	0.25	-2.94	[-3.48 - -2.39]	<0.001	-0.37	[-1.06 - 0.31]	0.28
Tripura	-0.01	[-0.26 - 0.23]	0.91	-0.83	[-2.16 - 0.51]	0.23	0.76	[-0.44 - 1.96]	0.22	-0.86	[-1.60 - -0.13]	0.02	1.58	[0.57 - 2.60]	<0.001
Uttar Pradesh	-1.42	[-1.54 - -1.29]	<0.001	0.49	[-0.21 - 1.18]	0.17	-0.62	[-1.15 - -0.09]	0.02	-3.45	[-3.76 - -3.14]	<0.001	-0.76	[-0.95 - -0.56]	<0.001
Uttarakhand	-0.76	[-1.15 - -0.36]	<0.001	-1.25	[-3.36 - 0.86]	0.25	-0.10	[-1.24 - 1.03]	0.86	-0.86	[-1.41 - -0.31]	<0.001	-0.86	[-1.35 - -0.37]	<0.001
West Bengal	-0.48	[-0.66 - -0.29]	0.00	-1.58	[-2.55 - -0.62]	0.00	1.14	[0.43 - 1.86]	<0.001	-1.25	[-1.69 - -0.81]	<0.001	0.12	[-0.51 - 0.75]	0.71
Ladakh (UT)										-0.92	[-1.33 - -0.50]	0.00	-0.48	[-1.09 - 0.14]	0.13
Andaman & Nicobar Islands (UT)													-0.08	[-1.70 - 1.55]	0.93
Chandigarh(UT)													-0.44	[-2.37 - 1.50]	0.65
Dadra & Nagar Haveli And Daman & Diu (UT)													0.14	[-1.46 - 1.75]	0.86
Lakshadweep (UT)													-0.11	[-0.77 - 0.55]	0.73
Puducherry (UT)													-0.96	[-2.08 - 0.16]	0.09

Table S4b: Standardized Absolute Change (SAC) in Prevalence and 95% CI for Boy Child Marriage for States and Union Territories of India between 2006-2016, 2016-2021, and 2006-2021

	2006-2021			2006-2016			2016-2021		
	SAC	95% CI	p-value	SAC	95% CI	p-value	SAC	95% CI	p-value
All India	-0.33	[-0.39 - 0.27]	<0.001	0.37	[-0.46 - -0.29]	<0.001	-0.23	[-0.35 - -0.11]	<0.001
Andhra Pradesh	-0.15	[-0.28 - -0.01]	0.03	-0.32	[-0.45 - -0.20]	<0.001	0.20	[-0.12 - 0.53]	0.21
Arunachal Pradesh	-0.28	[-0.75 - 0.18]	0.23	0.19	[-0.41 - 0.78]	0.54	-1.22	[-2.18 - -0.27]	0.01
Assam	-0.07	[-0.26 - 0.12]	0.47	-0.27	[-0.52 - -0.01]	0.04	0.33	[0.09 - 0.56]	0.01
Bihar	-0.55	[-0.99 - -0.10]	0.02	-1.12	[-1.77 - -0.47]	<0.001	0.60	[0.32 - 0.88]	<0.001
Chhattisgarh	-0.49	[-0.83 - -0.15]	0.01	-0.42	[-1.09 - 0.26]	0.23	-0.65	[-1.61 - 0.31]	0.19
Goa	-0.17	[-0.26 - -0.08]	<0.001	0.46	[0.23 - 0.70]	<0.001	-1.44	[-1.82 - -1.05]	<0.001
Gujarat	0.04	[-0.27 - 0.34]	0.82	-0.51	[-0.97 - -0.05]	0.03	1.13	[0.53 - 1.72]	<0.001
Haryana	-0.40	[-0.67 - -0.12]	0.01	-0.74	[-1.13 - -0.34]	<0.001	0.28	[0.09 - 0.48]	<0.001
Himachal Pradesh	-0.04	[0.10 - 0.02]	0.21	0.23	[0.09 - -0.56]	0.16	-0.59	[-1.21 - 0.03]	0.06
Jammu & Kashmir	-0.20	[-0.45 - 0.05]	0.11	-0.39	[0.75 - -0.03]	0.04	0.17	[-0.13 - 0.48]	0.26
Jharkhand	-0.64	[-1.12 - -0.17]	0.01	-1.29	[-1.97 - -0.61]	<0.001	0.66	[0.16 - 1.15]	0.01
Karnataka	-0.26	[-0.42 - -0.10]	<0.001	-0.36	[-0.59 - -0.12]	<0.001	-0.06	[-0.39 - 0.26]	0.70
Kerala	0.00	[0.00 - 0.00]	<0.001	0.00	[0.00 - 0.00]	<0.001	-0.15	[-0.32 - 0.02]	0.08
Madhya Pradesh	-0.31	[-0.61 - -0.01]	0.04	-0.68	[-1.13 - -0.23]	<0.001	0.43	[-0.02 - 0.87]	0.06
Manipur	0.35	[-0.06 - 0.77]	0.10	-0.14	[-0.31 - 0.04]	0.12	1.34	[0.11 - 2.57]	0.03
Meghalaya	-0.27	[-0.69 - 0.14]	0.19	-0.77	[-1.25 - -0.28]	<0.001	0.71	[-0.06 - 1.49]	0.07
Mizoram	-0.52	[-1.00 - -0.04]	0.03	-0.42	[-1.14 - 0.30]	0.25	-0.72	[-1.29 - -0.15]	0.01
Nagaland	-0.13	[-0.25 - -0.01]	0.03	-0.23	[-0.39 - -0.07]	0.01	0.06	[-0.17 - 0.30]	0.59
Nct Of Delhi	-0.21	[-0.35 - 0.07]	0.00	-0.21	[-0.44 - -0.01]	0.06	-0.21	[-0.39 - -0.02]	0.03
Odisha	-0.09	[-0.35 - 0.16]	0.46	-0.16	[-0.53 - 0.20]	0.39	0.04	[-0.42 - 0.49]	0.87
Punjab	-0.45	[0.74 - -0.15]	<0.001	-0.25	[0.79 - -0.28]	0.35	-0.84	[1.59 - -0.09]	0.03
Rajasthan	-1.36	[-1.78 - -0.94]	<0.001	-2.19	[2.95 - -1.42]	0.00	0.29	[-0.68 - 1.26]	0.55
Sikkim	-0.28	[-0.75 - 0.18]	0.23	0.19	[-0.41 - 0.78]	0.54	-1.22	[-2.18 - -0.27]	0.01
Tamil Nadu	0.02	[-0.03 - 0.07]	0.38	0.29	[-0.08 - 0.66]	0.13	-0.52	[-1.27 - 0.24]	0.18
Telangana	0.00	[0.00 - 0.00]	<0.001	0.00	[0.00 - 0.00]	0.00	-0.69	[-1.12 - -0.27]	<0.001
Tripura	-0.12	[-0.35 - 0.11]	0.29	-0.11	[-0.52 - 0.29]	0.59	-0.15	[-0.58 - 0.29]	0.51
Uttar Pradesh	-0.63	[-0.78 - -0.47]	<0.001	-1.04	[-1.39 - -0.70]	0.00	0.21	[-0.35 - 0.76]	0.47
Uttarakhand	-0.06	[-0.25 - 0.13]	0.51	0.30	[0.00 - -0.60]	0.05	-0.79	[-1.26 - -0.33]	<0.001
West Bengal	-0.08	[-0.28 - 0.12]	0.44	0.09	[-0.18 - 0.35]	0.53	-0.41	[-0.87 - 0.05]	0.08
Ladakh (UT)							-0.15	[-0.60 - 0.31]	0.52
Andaman & Nicobar Islands (UT)							-0.16	[-0.38 - 0.07]	0.18
Chandigarh (UT)							-0.59	[-0.92 - -0.25]	<0.001
Dadra & Nagar Haveli And Daman & Diu (UT)							-0.15	[-1.68 - 1.37]	0.84
Lakshadweep (UT)							-0.20	[-1.25 - 0.86]	0.71
Puducherry (UT)							-0.77	[-0.95 - -0.59]	0.00

Table S5: Estimated Headcount *N* boy child marriage for India and 36 States/Union Territories, and Percentage Share of each State/Union Territory to All-India per Indian legal Definition*, 2021

**All India**	Headcount	Percentage Distribution
	7,773,767	100
Bihar	1,354,551	17·42
West Bengal	1,308,086	16·83
Gujarat	919,913	11·83
Uttar Pradesh	755,014	9·71
Maharashtra	671,981	8·64
Rajasthan	435,154	5·60
Andhra Pradesh	380,106	4·89
Assam	361,982	4·66
Madhya Pradesh	327,797	4·22
Telangana	319,500	4·11
Karnataka	174,547	2·25
Jharkhand	131,064	1·69
Chhattisgarh	87,442	1·12
Haryana	77,091	0·99
Odisha	71,507	0·92
Jammu & Kashmir	64,215	0·83
Punjab	59,716	0·77
Tamil Nadu	55,498	0·71
NCT Of Delhi	42,310	0·54
Meghalaya	35,656	0·46
Tripura	34,461	0·44
Himachal Pradesh	22,213	0·29
Uttarakhand	19,688	0·25
Manipur	19,304	0·25
Kerala	12,482	0·16
Goa	8,684	0·11
Nagaland	5,043	0·06
Arunachal Pradesh	4,788	0·06
Mizoram	4,561	0·06
Dadra & Nagar Haveli	2,884	0·04
Sikkim	2,810	0·04
Ladakh	1,601	0·02
Andaman & Nicobar Island	1,177	0·02
Puducherry	941	0·01
Chandigarh	0	0·00
Lakshadweep	0	0·00

***Note:** Boy child marriage per the Indian legal definition is defined as boys married prior to their 21st birthday. We calculate estimates among men aged 23-27 years of age for consistency with the international definition.

Table S6: National Policies and Programs, and ratified international conventions that directly or indirectly influence child marriage in India

Policy Name	Year	Background	Source
**Policies directly targeted at Child Marriage**			
**Child Marriage Restraint Act**	1929	Any man under 21 and woman under 18 was considered as child; child marriage means if any of the spouse is a child. The penal provisions under the act do not invalidate the marriage, but may lead to simple imprisonment for fifteen days (extendable to three months) or fine of one thousand rupees or both. "Gauna" was not considered as child marriage	https://wcd.nic.in/child-marriage-restraint-act-1929-19-1929
**CHILDLINE**	1996	For any child in distress. Usually for street. Can dial 1098	https://web.umang.gov.in/landing/department/childline-1098.html
**Prevention of Child Marriage Act**	2006	It extends to whole of India (except J&K)- any male below 21 and female below 18 years of age is a child. Punishment for male marrying a child bride- rigorous imprisonment up to two year or fine of one lakh rupees or both. Same for anyone who solemnises such child marriage and the legal guardian of such child	https://indiankanoon.org/doc/790355/
**Protection of children from sexual offenses act**	2012	Defines child as anyone under the age of 18. Provides protection against sexual assault, sexual harassment. Sexual intercourse with child spouse comes within the purview of IPC and POSCO both.	https://vikaspedia.in/education/policies-and-schemes/protection-of-children-from-sexual-offences-act

**Prevention of Child Marriage (Amendment) Bill**	2021	The bill seeks to raise the age of marriage to 21 for women. The bill also seeks to make consequential amendments to other enactments about marriage such as the Indian Christian Marriage Act, The Hindu marriage act, 1955, the special marriage etc., 1954 etc.	https://pib.gov.in/PressReleasePage.aspx?PRID=1908007#:~:text=Further%2C%20the%20Governm
**Policies that indirectly impact child marriage**			
**Indian Christians Marriage Act**	1872	No bar on age. Therefore, has a negative impact	https://www.indiacode.nic.in/bitstream/123456789/2186/1/a1872-15.pdf
**Muslim Personal Law**	1937	No bar on age. Therefore, has a negative impact	https://indiankanoon.org/doc/1325952/
**Hindu Marriage Act**	1955	No bar on age. Therefore, has a negative impact	https://www.indiacode.nic.in/handle/123456789/1560?locale=en
**The Dowry Prohibition Act**	1961	Amended in 1986. The act prohibits the giving or taking of dowry that is any property or valuable security or agreed to be given either directly or indirectly by any party or parents of wither side at the time of marriage, or before or after the marriage ceremony. As child brides need low dowry, dowry prohibition will encourage lower rates of child marriage	http://www.nihfw.org/Legislations/THEDOWRYPROHIBITIONACT.html#:~:text=An%20Act%20to%20prohibit%20the,or%20after%20the%20marriage%20ceremony.
**Balika Samriddhi Yojana**	1997	Provides financial entitlements to BPL families for girl child below the age of 18 for their education and maintenance. As girls are married of earlier due to	https://vikaspedia.in/education/policies-and-schemes/scholarships/pre-

		financial burden, this policy will indirectly delay their age at marriage	metric-scholarships/balika-samridhi-yojana-bsy
**National Population Policy**	2000	Focuses on reducing population growth. One of its core goals is to delay marriage of girls, not before 18 years and preferably after 20 years.	https://meghealth.gov.in/dhs_mchfw_npp.html
**National Policy for Empowerment of Women**	2001	Includes objective of eliminating early marriages for girls	https://wcd.nic.in/womendevelopment/national-policy-women-empowerment
**National (and State) Commission for Protetcion of Child Rights**	2007	Aims to ensure that all laws, policies, programs and administrative systems conform to the vision of the rights of the child as enunciated in Constitution of India and the United Nations Convention on the Rights of the Child- any person below the age of 18. Therefore, the Commission plays a vital role in implementation of laws against Child Marriage	https://ncpcr.gov.in/about-ncpcr
**Rajiv Gandhi Scheme for Empowerment of Adolescent Girls (SABLA)**	2011	The scheme Sabla aims at empowering Adolescent Girls (AGs) (11-18 years) through nutrition, health care and life skills education. The non health component of the scheme that focuses on providing life and vocational skills to adolescent girls will help them to increase their employability. Increasing female employment and education is one of the pathways to increase female age at marriage	https://pib.gov.in/newsite/PrintRelease.aspx?relid=133064
**Rashtriya Kishor Swasthya Karyakram**	2014	One of the essential goals of RKSK is to reduce teenage pregnancies and therefore child marriages	https://vikaspedia.in/health/nrhm/national-health-programmes-

			1/rashtriya-kishor-swasthya-karyakram-rksk
**Beti Bachao Beti Padhao**	2015	Includes multisectoral interventions throughout the country. Education f the girls child is the essential component that will indirectly influence the age at marriage among girls	https://wcd.nic.in/bbbp-schemes
**National Plan of Action for Children**	2016	The plan aims at purposeful convergence and strong coordination across different sectors and levels of governance. It proposes comprehensive and actionable implementation, monitoring an evaluation framework through establishment of a National Co-ordination and Action Group (NCAG) to be headed at the level of Minister, Women and Child Development	https://wcd.nic.in/sites/default/files/National%20Plan%20of%20Action%202016.pdf
**National Policy for Women (draft)**	2016	While the goal of the policy is all-round development of females, particular objectives of "transforming discriminatory societal attitudes" and "a holistic life cycle approach" to women's health are closely related to child marriage; besides the other objectives of education and employment will help increase the age at marriage among girls	https://wcd.nic.in/sites/default/files/draft%20national%20policy%20for%20women%202016_0.pdf
**National Youth Policy**	2021	Seeks to catalyse widespread action on youth development across five priority areas, viz, education, employment and entrepreneurship, youth leadership and development, health, fitness and sports, and social justice. The components of social justice,	https://static.pib.gov.in/WriteReadData/specificdocs/documents/2022/may/doc20225553401.pdf

		education and employment will aid in reducing child marriage	
**Nari Adalat**	2021	It is a part of Mission Shakti that will provide alternate Grievance redressal system for women run by women. It can serve towards identifying and reducing cases of child marriages	
**International Conventions (ratified by India)**			
**Convention on the Elimination of All Forms of Discrimination Against Women (CEDAW)**	1979	It is an international legal instrument that requires countries to eliminate discrimination against women and girls in all areas and promotes women's and girls' equal rights	https://www.unwomen.org/en/digital-library/publications/2016/12/cedaw-for-youth#:~:text=The%20Convention%20on%20the%20Elimination,women's%20and%20girls'%20equal%20rights.
**Convention on the Right of the Child**	1990	Created to help children finally get the health care and nutrition they need to survive and develop, and there are stronger safeguards in place to protect children from violence and exploitation	https://www.unicef.org/child-rights-convention#learn
