# Supplementary material for: Prevalence of girl and boy child marriage across states and Union Territories in India, 1993–2021: a repeated cross-sectional study
Source: Lancet Glob Health. 2023 Dec 15;12(2):e271–81. doi: 10.1016/S2214-109X(23)00470-9 (PMC10805006; doi:10.1016/S2214-109X(23)00470-9)
Supplement: Equitable Partnership Declaration [file mmc2.pdf]

THE LANCET
Global Health

Supplementary appendix 2

This Equitable Partnership Declaration (EPD) was submitted by the authors, and we reproduce it as supplied. It has not been peer reviewed. *The Lancet*'s editorial processes have not been applied to the EPD.

Supplement to: Gausman J, Kim R, Kumar A, Ravi S, Subramanian SV. Prevalence of girl and boy child marriage across states and Union Territories in India, 1993–2021: a repeated cross-sectional study. *Lancet Glob Health* 2023; published online Dec 15. https://doi.org/10.1016/S2214-109X(23)00470-9.

Equitable Partnership Declaration questions

Researcher considerations

1. Please detail the involvement that researchers who are based in the region(s) of study had during a) study design; b) clinical study processes, such as processing blood samples, prescribing medication, or patient recruitment; c) data interpretation; and d) manuscript preparation, commenting on all aspects. If they were not involved in any of these aspects, please explain why.

   This question is intended for international partnerships; if all your authors are based in the area of study, this question is not applicable.

   This should include a thorough description of their leadership role(s) in the study. Are local researchers named in the author list or the acknowledgements, or are they not mentioned at all (and, if not, why)? Please also describe the involvement of early career researchers based in the location of the study. Some of this information might be repeated from the Contributors section in the manuscript. Note: we adhere to ICMJE authorship criteria when deciding who should be named on a paper.

**a) Study design:**
**b) Clinical study processes:**
**c) Data interpretation:**<br>Shamika Ravi, PhD (Economic Advisory Council to Prime Minister, Government of India) contributed to the data interpretation.
**d) Manuscript preparation:**<br>Shamika Ravi, PhD (Economic Advisory Council to Prime Minister, Government of India) provided critical review & editing of the draft.

2. Were the data used in your study collected by authors named on the paper, or have they been extracted from a source such as a national survey? ie, is this a secondary analysis of data that were not collected by the authors of this paper. If the authors of this paper were not involved in data collection, how were data interpreted with sufficient contextual knowledge?

   The Lancet Global Health *believe contextual understanding is crucial for informed data analysis and interpretation.*

Secondary analysis of National Family Health Surveys data was performed. The authors of this paper were not involved in data collection. All authors of this paper have substantial expertise and experience in analysing and interpreting the NFHS data. Shamika Ravi, PhD (Economic Advisory Council to Prime Minister, Government of India) contributed to the data interpretation and critical review & editing of the draft.

3. How was funding used to remunerate and enhance the skills of researchers and institutions based in the area(s) of study? And how was funding used to improve research infrastructure in the area of study?

   Potentially effective investments into long-term skills and opportunities within institutions could include training or mentorship in analytical techniques and manuscript writing, opportunities to lead all or specific aspects of the study, financial remuneration rather than requiring volunteers, and other professional development and educational opportunities.

   Improvements to research infrastructure could be funding of extended trial designs (such as platform trials) and use of master protocols to enable these designs, establishment of long-term contracts for research staff, building research facilities, and local control of funding allocation.

**Skills:**
**Research infrastructure:**<br>Funding from Bill & Melinda Gates Foundation INV-002992 is being used to create an interactive dashboard with health and population data modelled at various geographic levels.

4. How did you safeguard the researchers who implemented the study?

   Please describe how you guaranteed safe working conditions for study staff, including provision of appropriate personal protective equipment, protection from violence, and prevention of overworking.

This study involved secondary analysis with minimal risk to researchers.

<u>Benefits to the communities and regions of study</u>

5. How does the study address the research and policy priorities of its location?

   How were the local priorities determined and then used to inform the research question? Who decided which priorities to take forward? Which elements of the study address those priorities?

All authors of this paper agreed the following as important local priorities. First, the insight we provide into the subnational variation in child marriage in India is not only directly relevant to achieving SDG #5.3.1, which calls to eliminate girl child marriage by 2030, but also will inform current legislative and policy debate in India regarding the country's legal age at marriage. Second, our paper includes estimates of boy child marriage, which has largely been overlooked and is now becoming recognized as a global priority. Third, our paper uses rigorous methodologies to make geographic delineations of India in previous years the same as the administrative

configuration in 2021, which can be used in future research to assess progress in other key health metrics in India at the state/UT level over time.

6. How will research products be shared in the community of study?

For instance, will you be providing written or oral layperson summaries for non-academic information sharing? Will study data be made available to institutions in the region(s) of study? The Lancet Global Health *encourages authors to translate the summary (abstract) into relevant languages after paper editing; do you intend to translate your summary?*

For non-academic information sharing, we developed an interactive view of the state maps and data via a dashboard: https://geographicinsights.iq.harvard.edu/State-Child-Marriage.

7. How were individuals, communities, and environments protected from harm?

a) *How did you ensure that sensitive patient data was handled safely and respectfully? Was there any potential for stigma or discrimination against participants arising from any of the procedures or outcomes of the study?*

Not applicable.

b) *Might any of the tests be experienced as invasive or culturally insensitive?*

Not applicable.

c) *How did you determine that work was sensitive to traditions, restrictions, and considerations of all cultural and religious groups in the study population?*

Not applicable.

d) *Were biowaste and radioactive waste disposed of in accordance with local laws?*

Not applicable.

e) *Were any structures built that would have impacted members of the community or the environment (such as handwashing facilities in a public space)? If so, how did you ensure that you had appropriate community buy-in?*

Not applicable.

f) *How might the study have impacted existing health-care resources (such as staff workloads, use of equipment that is typically employed elsewhere, or reallocation of public funds)?*

Not applicable.

8. Finally, please provide the title (eg, Dr/Prof, Mr/Mrs/Ms/Mx), name, and email address of an author who can be contacted about this statement. This can be the corresponding author.

Name: Dr. S V Subramanian
Email: svsubram@hsph.harvard.edu
